# School-Partnered Collaborative Care (SPACE) for Pediatric Type 1 Diabetes: Development and Usability Study of a Virtual Intervention With Multisystem Community Partners

**DOI:** 10.2196/64096

**Published:** 2025-03-26

**Authors:** Christine A March, Elissa Naame, Ingrid Libman, Chelsea N Proulx, Linda Siminerio, Elizabeth Miller, Aaron R Lyon

**Affiliations:** 1 Pediatric Endocrinology UPMC Children's Hospital of Pittsburgh Pittsburgh, PA United States; 2 Department of Pediatrics University of Pittsburgh Pittsburgh, PA United States; 3 Clinical and Translational Science Institute University of Pittsburgh Pittsburgh, PA United States; 4 Department of Medicine University of Pittsburgh Pittsburgh, PA United States; 5 Adolescent and Young Adult Medicine UPMC Children's Hospital of Pittsburgh Pittsburgh, PA United States; 6 Department of Psychiatry and Behavioral Sciences University of Washington Seattle, WA United States

**Keywords:** type 1 diabetes, user-centered design, school health, collaborative care model, implementation research, pediatric, usability testing, virtual intervention, multisystem community partners, children, youth, diabetes management support, health system partners, psychosocial interventions, quantitative assessments, qualitative assessments

## Abstract

**Background:**

School-partnered interventions may improve health outcomes for children with type 1 diabetes, though there is limited evidence to support their effectiveness and sustainability. Family, school, or health system factors may interfere with intervention usability and implementation.

**Objective:**

To identify and address potential implementation barriers during intervention development, we combined methods in user-centered design and implementation science to adapt an evidence-based psychosocial intervention, the collaborative care model, to a virtual school-partnered collaborative care (SPACE) model for type 1 diabetes between schools and diabetes medical teams.

**Methods:**

We recruited patient, family, school, and health system partners (n=20) to cocreate SPACE through iterative, web-based design sessions using a digital whiteboard (phase 1). User-centered design methods included independent and group activities for idea generation, visual voting, and structured critique of the evolving SPACE prototype. In phase 2, the prototype was evaluated with the usability evaluation for evidence-based psychosocial interventions methods. School nurses reviewed the prototype and tasks in cognitive walkthroughs and completed the Intervention Usability Scale (IUS). Two members of the research team independently identified and prioritized (1-3 rating) discrete usability concerns. We evaluated the relationship between prioritization and the percentage of nurses reporting each usability issue with Spearman correlation. Differences in IUS scores by school nurse characteristics were assessed with ANOVA.

**Results:**

In the design phase, the partners generated over 90 unique ideas for SPACE, prioritizing elements pertaining to intervention adaptability, team-based communication, and multidimensional outcome tracking. Following three iterations of prototype development, cognitive walkthroughs were completed with 10 school nurses (n=10, 100% female; mean age 48.5, SD 9.5 years) representing different districts and years of experience. Nurses identified 16 discrete usability issues (each reported by 10%-60% of participants). Two issues receiving the highest priority (3.0): ability to access a virtual platform (n=3, 30% of participants) and data-sharing mechanisms between nurses and providers (n=6, 60% of participants). There was a moderate correlation between priority rating and the percentage of nurses reporting each issue (ρ=0.63; *P*=.01). Average IUS ratings (77.8, SD 11.1; 100-point scale) indicated appropriate usability. There was no difference in IUS ratings by school nurse experience (*P*=.54), student caseload (*P*=.12), number of schools covered (*P*=.90), or prior experience with type 1 diabetes (*P*=.83), suggesting that other factors may influence usability. The design team recommended strategies for SPACE implementation to overcome high-priority issues, including training users on videoconferencing applications, establishing secure forms for school data reporting, and sharing glucose data in real-time during SPACE meetings.

**Conclusions:**

Cross-sector interventions are complex, and perceived usability is a potential barrier to implementation. Using web-based cocreation methods with community partners promoted high-quality intervention design that is aligned with end-user priorities. Quantitative and qualitative assessments indicated appropriate degree of usability to move forward with pilot-testing.

## Introduction

Supportive parent and peer relationships can have a significant impact on diabetes-related behaviors, glycemia, and psychosocial outcomes of children and adolescents with type 1 diabetes [1-4]. For this reason, parent and peer interactions have been the target of numerous community-based interventions [5-7]. There are other natural support systems in the community for children with type 1 diabetes and their families. In particular, schools serve a critical role in the development of children. School success is linked to professional attainment and health in adulthood, making education an important social determinant of health [8]. For children with type 1 diabetes, attendance at in-person schools may benefit diabetes management practices through establishing routines for meal timing and physical activity [9,10]. Children with diabetes are also supported by numerous legal protections to ensure they have appropriate medical care in school and a safe learning environment [11].

Despite the role of schools, there are ongoing challenges with diabetes care there. School nurses have identified gaps in their diabetes training, particularly related to new diabetes technologies [12,13]. This can adversely affect both student and parent experiences with school care [14-16] and may impact health outcomes, as young children with type 1 diabetes have higher blood glucose on average during school as compared to weekends or virtual school days [17]. School nurses have also endorsed difficulty coordinating care with students’ medical teams, which can lead to gaps in care [12]. Pediatric diabetes providers understand the importance of school-based diabetes care, though they have similarly reported challenges interfacing with schools due to gaps in school staff education, lack of awareness of specific policies, and poor systems for communication [18]. Interventions to address these challenges in the school setting have been limited in scope and impact [19], and different barriers may hamper joint interventions. At the school level, there may be competing priorities between health and educational initiatives, the confines of the school day, and staffing or resource limitations, driven by state-level policies and funding. Health systems similarly encounter challenges with staffing and resources, which impair the ability to communicate with and train school health staff [18]. Enhancing partnerships through collaborative health service interventions may improve diabetes care in the school setting [20].

To bridge the school-provider practice gap, the objective of this study was to develop a school-partnered collaborative care (SPACE) model for pediatric type 1 diabetes to bring together schools, health care providers, and families into a comprehensive diabetes care team using digital technologies. SPACE was modified from the collaborative care model (CCM), an evidenced-based, integrated care model for pediatric and adult mental health care with several core components [21]. A CCM classically partners multidisciplinary teams with a care manager (core component: patient-centered care team). The team regularly screens candidates for the program (core component: population-based care), develops a shared treatment plan, tracks progress with valid measures (core component: measurement-based treatments to target), and makes treatment recommendations in a stepwise approach (core component: evidence-based care). Originally used in the primary care setting, the CCM has been associated with improved outcomes in youth with depression [22] and in adults with combined depression and chronic illness, including poorly controlled diabetes [23]. The CCM has been adapted for the school setting, as schools are uniquely positioned to identify at-risk students, offer services, and treat co-occurring academic problems [24,25]. The CCM is well-suited for school-based diabetes care, as it could be used to better connect school personnel with diabetes medical teams to overcome barriers in communication and identify and address opportunities to improve diabetes management by integrating a diabetes expert into the school health team.

Translating the CCM to type 1 diabetes required modifications to both content (related to the diagnosis) and contextual factors (local school setting). To accomplish this, we relied on user-centered design (UCD), a field that is relatively new to the health sciences [26], in combination with concepts from implementation science (IS) [27]. The goals of UCD are to promote the development of interventions that are easy to learn, efficient, acceptable, sustainable, and most importantly, fit to the local context [28]. UCD draws from a multidisciplinary background in human-computer interaction, industrial design practices, cognitive psychology, and participatory research [28]. In this application, UCD involves a set of procedures to cocreate interventions with the individuals who will ultimately use them [29]. UCD can be strengthened by combining it with theories, frameworks, and models drawn from IS [27]. Merging methods from UCD and IS can enable investigators to simultaneously assess multilevel barriers and facilitators which may influence implementation during intervention development. Investigators may also work with design teams to select implementation strategies for future testing or incorporation into clinical practice. In this study, integrating UCD and IS strategies was innovative and essential, as the modifications required navigation of two complex ecosystems, schools, and an academic diabetes medical center. In this paper, we present SPACE design activities and assessments of usability, an indicator of design quality [30], and a determinant affecting intervention feasibility and acceptability [30,31], with target end users (school nurses).

## Methods

### Study Design

**Figure 1:**
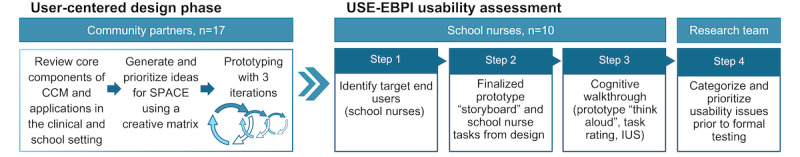
Overview of the cocreation methods and usability testing to design the SPACE intervention. In the first phase, we used user-centered design strategies to generate iterative prototypes of the intervention with multisystem community partners. In the second phase, we adopted the USE-EBPI methods to assess usability with target end users (school nurses). CCM: collaborative care model; SPACE: school-partnered collaborative care; IUS: Intervention Usability Scale; USE-EPBI: usability evaluation for evidence-based psychosocial interventions.

We used cocreation methods to design SPACE and assess preliminary usability prior to full-scale implementation [32]. The goal of the SPACE adaptation was to maintain fidelity to the core components of the CCM with the addition and removal of some elements to accommodate the differing content and contextual factors [33]. All modifications were proactive and preplanned prior to full-scale implementation. A summary of processes is depicted in Figure 1. The research team overseeing all activities was comprised of four physicians, a nurse, a psychologist, and a UCD consultant. Together, this team had expertise in type 1 diabetes clinical care, type 1 diabetes school care, school-based health services and research, UCD methods, and IS. All UCD activities were facilitated by a trained investigator in UCD (CAM) with input from other team members. No member of the research team had a diagnosis of or child with type 1 diabetes, though one physician (EM) had a role as the medical doctor for a local school district.

### Design Strategies

We iteratively adapted the CCM to create SPACE with a design team of community partners with a vested interest in school-based diabetes care. Roles were identified through stakeholder mapping with an established research advisory board [34]. We recruited partners from three primary groups to represent schools, patients and families, and health systems. School partners included school nurses, educators, and administrators with current working experience with children with type 1 diabetes. Patient and family partners included individuals with type 1 diabetes for ≥6 months, parents of children with type 1 diabetes for ≥6 months, and community advocates. Health system partners included specialists who manage children with type 1 diabetes and paraprofessionals who interact with school systems (eg, nurses, diabetes care and education specialists, and social workers). Partners could identify with more than one role. All partners were required to reside or be employed in our geographic region (Pennsylvania) and participate in English. An established research advisory board served as the foundation for the design team; additional members were recruited through the research team’s existing relationships with the Pennsylvania Association of Nurses and Practitioners, our diabetes center, and local branches of national diabetes advocacy organizations. To manage potential power differentials that can exist between these roles [35], we used three strategies: (1) participants completed basic training in ethical research [36], (2) design meetings began by recognizing the importance of each role’s unique contributions, and (3) meetings involved a combination of individual and group activities to limit influence from any one person’s ideas.

The research team held a series of three monthly 90-minute design meetings. Design meetings were web-based using a videoconferencing platform, Zoom (Zoom Video Communications), which could be accessed by phone, tablet, or computer. The research team met with each community partner either individually or in a group setting to ensure they had access to the videoconferencing platform. All partners were trained on a shared digital whiteboard (Mural Visual Collaboration) to enable active participation in meetings. Two meeting time options were offered each month to increase flexibility and maximize the involvement of all community partners. Partners were provided with meeting agendas in advance, as well as relevant materials to review if able.

Each meeting served as an iterative design cycle for a total of three cycles [37]. The activities generated an intervention prototype and potential strategies for future implementation. The research team provided three assumptions to ground group ideation: (1) the SPACE team must include the school nurse, family, and diabetes medical team at a minimum, (2) the intervention will be geared toward younger children (6 to 13 years of age) who are more likely to rely on a school nurse, and (3) all SPACE activities would be virtual to engage school districts within our broader region and fit within the diabetes medical team’s workflow. Each cycle included bidirectional sharing of information between the research team and partners. In the first meeting, we reviewed the core components and evidence for the CCM with examples from the literature of the CCM being used in clinical and school settings. Partners independently generated ideas for the adaptation based on the CCM components and roles involved using a creative matrix [38]. In the matrix, the column headings identified the participating role (eg, student, parent, school nurse, diabetes medical team, or other), and the row headings identified important features of SPACE (engagement, structure and content, outcomes, supports and policies, or other). Partners categorized ideas by where they best fit, acknowledging some ideas may bridge between multiple roles or concepts. Following a brief discussion of each idea, partners used a visual voting system to identify first and secondary priorities for the CCM, generating a semiquantitative indicator for each idea. The research team assigned two points for each first-priority vote and one point for each second-priority vote. The total points for each idea were summed.

In the subsequent two cycles, the research team presented increasingly detailed versions of the prototype, with the end goal being a narrative storyboard representing the SPACE intervention. At each stage, the facilitator asked partners to reflect on SPACE to identify strengths, limitations, opportunities for refinement, and areas in need of further clarity. In the final session, partners also discussed the individual tasks school nurses would be responsible for in SPACE to identify which tasks should be targeted for user testing.

### Usability Assessment

We adopted the usability evaluation for evidenced-based psychosocial interventions (USE-EBPI) to evaluate usability, which allows for the discovery and organization of potential barriers and planning for strategies to overcome them [39]. Usability was assessed both quantitatively and qualitatively through cognitive walkthroughs with school nurses (n=10). The USE-EBPI methods outline four steps, including identifying users for testing, defining EBPI tasks, conducting the evaluation, and organizing and prioritizing usability issues. First, we identified that school nurses were appropriate end users for testing (step 1), with the sample size determination based on usability modeling [40,41]. School nurses were recruited through the email listserv of the Pennsylvania Association of School Nurses and Practitioners, which is the state branch of the National Association of School Nurses. School nurses in Western Pennsylvania, where this intervention will be formally pilot-tested, were preferentially recruited. School nurses who participated in the original SPACE design were excluded to allow for a more objective assessment. The prototype and associated tasks for testing were identified through the design process with community partners (step 2). Cognitive walkthroughs were conducted using a videoconferencing platform (step 3). Each session lasted approximately 45 minutes and was attended by two members (CAM and EN) of the research team for facilitation and detailed note-taking. All sessions were audio-recorded to allow for review to ensure all ideas were captured.

Each cognitive walkthrough had two components. First, school nurses were led sequentially through each step of SPACE using the storyboard prototype and asked to think aloud about the intervention. Subsequently, we presented the school nurses with nine case scenarios describing intervention tasks. For each case scenario, school nurses were asked to provide a rating for how likely they would be able to do the task and a justification. Ratings used a 5-point Likert-type response scale with 1 indicating no or very small chance of success and 5 indicating a very good chance of success. Detailed notes were taken throughout the cognitive walkthrough. Subsequently, school nurses completed the Intervention Usability Scale (IUS), a 10-item, validated survey that is used as a benchmark in intervention redesign [42]. The IUS has strong psychometric properties including a two-factor solution (“usable” and “learnable”) and a Cronbach α of 0.83 in a sample of medical professionals [42]. A benchmark IUS score of >70 (range 0-100) corresponds to an acceptable level of usability [43].

### Data Management and Analysis

Qualitative notes from the cognitive walkthroughs were typed, deidentified, and reviewed weekly by two members of the research team (EN and CAM). Usability issues captured from the notes related to both the intervention generally and its specific components, as elicited by the scenarios. The reviewers tallied the number of participants who identified the same issue, adding new usability issues as needed as cognitive walkthroughs continued. Once completed, the reviewers organized the usability issues by type using 13 categories in the UCD literature (step 4) [30]. Two investigators assigned a priority score (1=not important, 2=somewhat important, and 3=very important) for additional adaptations needed to generate a workable intervention. Priority scores were based on the perceived likely impact on future end users, the likelihood that this would be experienced by users, and how critical it is for the success of SPACE [39]. Independent scores were then averaged and sorted from highest to lowest priority. We examined the correlation between the priority rating and the percentage of school nurse participants identifying the issue using Spearman’s correlation. The usability issues and priority rankings were shared with the design team to determine any additional refinements to SPACE.

Quantitative data included case scenario ratings and IUS scores. Ratings for each case scenario and the IUS scores were averaged across participants and presented as a mean and SD. We explored differences in IUS scores using one-way ANOVA among groups with differing characteristics perceived to influence school nurse workload and skill level, including school nursing experience (<10 years vs ≥10 years), caseload (<750, 750-1000, or 1001-1500 students), number of schools covered (1, 2, and more than 2), and students with type 1 diabetes in the past 5 years (<5, 5-10, or >10 students) [44]. A *P* value of <.05 was considered significant. All statistical analyses were completed using StataSE (version 17; StataCorp).

### Ethical Considerations

All design and research activities were deemed exempt by the University of Pittsburgh Institutional Review Board (PRO 23110009). As the study was exempt, we were not required to document written informed consent. All research participants were presented with a consent script describing the purpose of the study, study activities, compensation, risks, and benefits. Verbal consent was obtained. All study data were deidentified and linked to private identifiable information using a unique code. Community partners were compensated US $25 per hour (US $37.50 per 90-minute meeting), for a total of US $150. Compensation was provided for partners who could not attend a meeting if they reviewed materials and provided feedback via phone or email. School nurses participating in usability tests were compensated US $30.

## Results

### Overview

We recruited 20 community partners for the design team. Three community partners were unable to attend the meetings due to changes in their family circumstances. The remaining 17 community partners reflected all intended roles (Table 1). The school nurses were employed in rural, urban, and suburban school districts of different sizes. A total of 3 (18%) partners had a personal diagnosis of type 1 diabetes, giving them the additional role as a patient. Monthly attendance ranged from 15 (88%) to 17 (100%) participants. Personal communications were used to follow up with any partner who could not attend a scheduled meeting.

**Table 1:** SPACE^a^ design team community partner roles (n=17).

Type of partner		Value^b^, n (%)
**School**		8 (47)
	School nurse	4 (24)
	Administrator or educator	4 (24)
**Patient or family**		8 (47)
	Individual with diabetes	3 (18)
	Parents	4 (24)
	Community advocate	1 (6)
**Health system**		6 (35)
	Diabetes specialists	2 (12)
	Diabetes care and education specialist	1 (6)
	Social workers	2 (12)
	School nurse navigator	1 (6)

^a^SPACE: school-partnered collaborative care.

^b^Numbers add to more than 17 as partners could identify with more than one role.

### Intervention Design

At the initial design meeting, participants generated 141 ideas for the SPACE redesign, of which 94 were unique. Partners assigned a numeric prioritization to ideas, which were then condensed to create a list of unique ideas (Multimedia Appendix 1). Higher prioritized design ideas by SPACE category and role from the creative matrix are summarized in Table 2. Many focused on flexibility in scheduling, data sharing and communication during and between meetings, multidimensional outcome tracking, and the team approach with other vested stakeholders. Other ideas included having students lead the SPACE discussions if developmentally appropriate (9 points) and having the school nurse identify barriers to the student’s diabetes management in school (8 points).

The research team used these ideas to generate an initial concept, functioning as a low-fidelity prototype, summarizing the SPACE intervention (Multimedia Appendix 2). The concept poster summarized the team members and roles, the structure of and topics addressed during SPACE meetings, and potential outcomes to track for the students. Partners provided a critique using individual text responses on the shared whiteboard, followed by group discussion, aligned with four categories in a feedback capture grid (strengths, limitations, opportunities, and questions; Textbox 1). Identified strengths focused on the ability for “everyone to share ideas at the same time” to streamline communication, give the school nurse personalized diabetes advice, and offer the family a team outside of the hospital. Partners also appreciated the flexibility of meeting scheduling and the emphasis on identifying specific goals that are measurable to help the student “feel good and motivated to move forward.” Constructive feedback identified potential challenges at the student, school nurse, and parent levels. For students, these may include the impact of meeting attendance on class time and the willingness to share if the team has too many members. For nurses and parents, these included finding a common time for both meetings and communication between meetings. Additional parent challenges included the financial burden of any recommended referrals and the reliance on disclosure to offer resources. To overcome these, the partners suggested maximizing flexibility in scheduling, offering resources to all parents regardless of disclosure, and determining team size based on student comfort level. Partners also suggested the SPACE intervention consider strategies to incentivize less engaged students and reach out to other school staff with educational activities about diabetes. Questions included how meetings would be conducted (eg, the virtual platform), follow-up documentation for team members, what to do if the parent or nurse does not come to the meeting, and what if any communication between visits should be required.

**Table 2:** Representative design ideas from the creative matrix exercise for the SPACE^a^ adaptation with associated point totals indicating prioritization^b^.

	Student	Parent	School nurse	Medical team
Engagement	Offer the student incentives (n=8) Establish criteria for student considered high risk and in need of more support (n=2)	Flexible scheduling (n=8) Review different parent motivations to participate (n=2)	Include a school administrator (n=4) Identify daytime coverage or availability (n=1)	Screen for social determinants of health (n=2) Identify patients at medical appointments (n=2)
Structure and content	Choose one thing to work on at a time (n=4) Address consistent topics (n=2)	Parent and school nurse communication plan (n=7)	Check in with student between meetings (n=8) School nurse contributes data (n=2)	Identify education needs of the parent, student, and nurse (n=8) Adjust written care plans (n=3) Referrals to other services (2)
Outcomes	Time in the classroom (n=11) Confidence in skills (n=8)	Communication with school nurse (n=2)	Assessment of self-management skills (n=7) Attendance (n=4)	Glycemia (n=14)
Supports and policies	Cooperation from peers (n=4)	Family support and collaboration (n=8) Person-friendly language (n=1)	Cooperation from teachers (4) Support from school administration (n=2)	Diabetes Medical Management Plan (n=6)

^a^SPACE: school-partnered collaborative care.

^b^Higher numbers indicate greater prioritization from the study team.

**Summary of partner critique to the school-partnered collaborative care (SPACE) concept or low-fidelity prototype.**

**Strengths**

- Multidisciplinary approach between school, family, and health care system
- Flexibility in scheduling for school and parent
- Focus on tangible outcomes for the student

**Limitations**

- Student concerns: missed class time, comfort with discussing diabetes in the group setting
- Parents: scheduling, reliance on disclosure to identify supportive resources, financial burden of referrals
- School nurses: scheduling, bandwidth to communicate with parents between meetings

**Opportunities**

- Strategies to incentivize students who are less engaged or experiencing burnout
- Parallel education programs for school staff

**Questions**

- Technical aspects (eg, What platform will be used to share information?)
- Follow-up documentation (eg, Who provides the follow-up calls or evaluation?)
- Meeting no-shows (eg, If key members cannot be at meetings, how will the info be communicated?)

We then generated a more detailed prototype of SPACE using a storyboard, narrating the intervention from the initial recruitment of a student through the first SPACE meeting. Partners were given an opportunity to review the prototype independently. During the meeting, partners were split into small groups to go through the prototype together. In addition to minor changes in word choice, this second review generated six areas in need of clarity: a reference to obtaining parental permission, the inclusion of teacher support when applicable, clarifying expectations for parental involvement, giving multiple examples for student diabetes goals, modifying language from barriers to factors which may positively or negatively influence diabetes goal attainment, and promoting changes to a 504 or other written accommodations plan. The final storyboard is included in Multimedia Appendix 3.

Partners identified nine scenarios or tasks for school nurse user testing, including securing protected time to participate, identifying candidate students, approaching families about participation, naming potential diabetes-related issues in school and factors contributing to these, selecting and recruiting additional team members, addressing mental health concerns, and listing activities that they can do with the student to work on diabetes habits between meetings. Recommended strategies to foster implementation in schools included leaning on existing programs (eg, the student assistance team in Pennsylvania) and offering incentives to the school district (designation or certification as a “SPACE” school) or school nurse (continuing education credits for participation).

### Usability Testing

**Figure 2:**
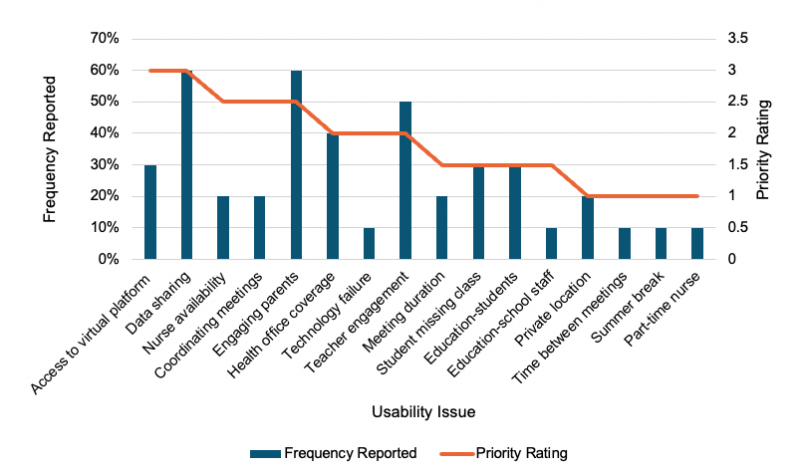
Relationship between the percentage of school nurses reporting each usability issue (bar chart) and priority ratings from the research team (line).

We recruited ten school nurses, each from a different school district in Western Pennsylvania, reflecting diverse experiences with diabetes care and school health (Table 3). School nurses identified many positive aspects of the SPACE model and 16 unique usability concerns. School nurses liked that the intervention offered a streamlined process to communicate with parents and health care providers and often found the time commitment to be realistic and manageable. Each usability issue was identified by between 10% and 60% of testers. The issues aligned with eight categories from Munson et al [30] related to intervention complexity, available time, workflow, existing infrastructure and resources, perceived value, trust between families and school nurses, and reliance on technology. Priority scores ranged from 1.0=lowest priority to 3.0=highest priority. Figure 2 visually displays the relationship between priority ratings and the frequency each issue is reported. There was a moderate correlation (ρ=0.63; *P*=.01) between priority rating and the percentage of school nurses reporting the issue. Two usability issues had the highest priority (3.0), including accessing the virtual platform and establishing a secure mechanism for data sharing between the school and health care provider (Table 4). Other higher-priority issues included coordinating meetings, nurse availability or health office coverage, and parent engagement. One issue, teacher or other staff engagement, was frequently reported but was deprioritized by the research team as it was less critical to the success of SPACE.

**Table 3:** Characteristics of school nurse participants (n=10) for usability tests.

Characteristic		Value
Female gender identity, n (%)		10 (100)
Age (years), mean (SD)		48.5 (9.5)
**Highest nursing degree, n (%)**		
	Bachelor’s degree	5 (50)
	Master’s degree or above	5 (50)
**School nursing experience (years), n (%)**		
	<10 years	3 (30)
	≥ 10 years	7 (70)
**Number of schools covered, n (%)**		
	1	4 (40)
	2	3 (30)
	More than 2	3 (30)
**Geographic setting, n (%)**		
	Rural	2 (20)
	Suburban	7 (70)
	Urban	1 (10)
**Grades covered^a^, n (%)**		
	Elementary school	7 (70)
	Middle school	6 (60)
	High school	8 (80)
**Student caseload, n (%)**		
	<750 students	3 (30)
	750-1000 students	2 (20)
	1001-1500 students	5 (50)
**Students with diabetes in the past 5 years, n (%)**		
	<5 students	4 (40)
	5-10 students	4 (40)
	>10 students	2 (20)

^a^School nurses could select more than one choice, so numbers total to greater than 100%.

**Table 4:** Summary of case scenario ratings and justifications.

Scenario	Scenario topic	Rating, mean (SD)	Example comments
1	Accommodate SPACE^a^ visits in nurse schedule	4.35 (0.67)	“I can block out time since this would be for a student, so the staff will cover me. The secretary might help with triage and will let teachers know I am busy. The only interruption would be in acute emergencies ... I’ve done this for students before.” [Nurse 1]
2	Identify students for SPACE	4.90 (0.32)	“We know our students and can identify who’s in need.” [Nurse 6]
3	Discuss SPACE with parents	4.55 (0.76)	“It’s still so new; I need to learn and experience it more to feel comfortable enough to explain it to parents and five all its benefits and value to engage them.” [Nurse 7]
4	Name diabetes-related goals for SPACE	4.65 (0.47)	“The school nurse is able to look at the medical aspect of blood glucoses, how they’re doing, treating, interacting with others, doing at school ... we can look at these areas and set a goal.” [Nurse 3]
5	Identify other school staff to participate	4.45 (0.69)	“I can look up their schedules and see who teaches the child, who they spend the most time with. I can also check in with the counselors; sometimes they may have a good rapport with the child and their presence would help.” [Nurse 2]
6	Approach other school staff to participate	4.50 (0.58)	“We’re doing it already with 504 plans.” [Nurse 9]
7	Discuss mental health concerns	4.60 (0.52)	“I’d do it. Mental health is essential.” [Nurse 10]
8	Identify barriers affecting goal attainment	4.58 (0.55)	“I’d put on a detective hat and go look!” [Nurse 5]
9	Develop strategies to coach student	4.38 (0.72)	“That’s what I do! This is where I can help educate families about how we do this.” [Nurse 4]

^a^SPACE: school-partnered collaborative care.

School nurses generally indicated a high likelihood of success in the nine case scenarios, with mean Likert scale scores ranging from 4.35 to 4.90 (Table 4). The scenario with the lowest score, scenario 1, related to accommodating the SPACE visits during the school day. Acknowledging the challenge of blocking time for meetings, school nurses identified different workarounds to make time for these meetings, which they commonly use for other types of meetings (eg, for 504 plans). Some suggested having the meeting immediately before or after school, arranging coverage with an administrative assistant or other staff, or spacing out visits for different students so they are not on the same day.

IUS scores ranged from 65.0 to 92.5, with an average score of 77.8 (SD 11.1), meeting our predetermined benchmark for acceptable usability [43]. In exploratory analyses, there was no relationship between IUS score and any school nurse characteristics, including years of school nursing experience, student caseload, number of schools covered, or number of students with diabetes in the past five years (Table 5).

**Table 5:** IUS^a^ scores by school nurse characteristics.

Characteristic			IUS score, mean (SD)		*P* value
**School nursing experience**					.54
	<10 years (n=3)	74.2 (15.9)			
	≥10 years (n=7)	79.3 (9.5)			
**Caseload**					.12
	<750 students (n=3)	83.3 (15.9)			
	750-1000 students (n=2)	86.3 (1.8)			
	1001-1500 students (n=5)	71.0 (5.8)			
**Number of schools**					.90
	1 (n=4)	79.4 (12.0)			
	2 (n=3)	75.0 (11.5)			
	More than 2 (n=3)	78.3 (13.8)			
**Students with diabetes in the past 5 years**					.83
	<5 students (n=4)	76.3 (9.2)			
	5-10 students (n=4)	80.6 (14.0)			
	>10 students (n=2)	75.0 (14.1)			

^a^IUS: Intervention Usability Scale.

**Figure 3:**
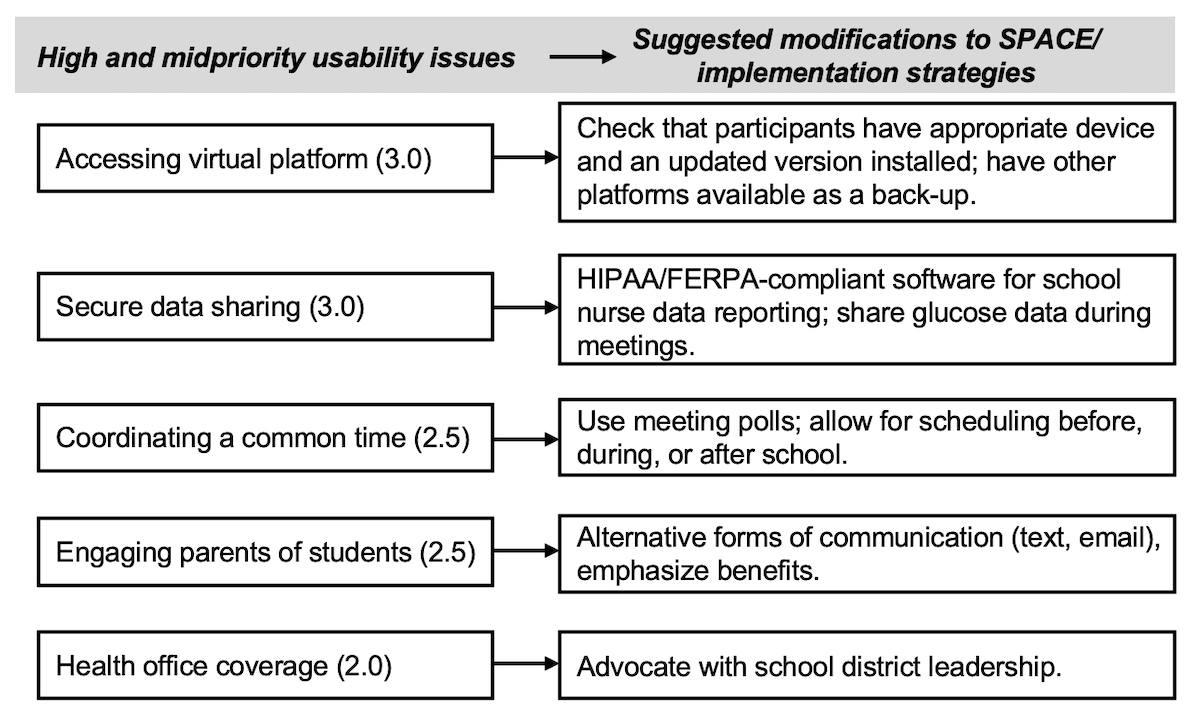
Design team recommendations for SPACE implementation based on high- and midpriority usability issues. FERPA: Family Educational Rights and Privacy Act; HIPAA: Health Insurance Portability and Accountability Act; SPACE: school-partnered collaborative care.

The usability issues were reviewed by the design team prior to implementation of the SPACE pilot, including high-priority issues (accessing the virtual platform, sharing data) and midpriority issues (coordinating a common time, ensuring health office coverage, and engaging parents). Several suggestions were made by the design team and subsequently implemented in the pilot, including both modifications to the intervention and strategies to implement it. A summary of specific strategies by usability issue is included in Figure 3.

## Discussion

### Principal Findings

School management of diabetes is highly important to the overall care of children with type 1 diabetes, yet school-based or school-partnered interventions remain understudied and underused. Interventions bridging the school and health systems are inherently complex, which may complicate their long-term use [45]. Our proposed modifications exhibited similar complexity, with multiple interacting components that may require organizational or workforce accommodations for implementation [46]. Our process was intended to overcome potential barriers using best practices in UCD [37]. The combination of UCD methods and usability testing with target end users enabled key stakeholders to guide all aspects of intervention design, promoting fitness for the school setting and establishing credibility and trust [27]. Furthermore, our web-based approach, including a shared whiteboard, enabled us to iteratively develop SPACE in a relatively short time with a diverse group of people who often have severe constraints on their time (eg, hospital and school staff). Finally, reviewing the potential usability issues during the design phase helped to refine the prototype in preparation for implementation.

The SPACE model is a fully developed intervention prototype that will bring together the school nurse, parent, and health care provider into a multidisciplinary care team to support students with type 1 diabetes in a structured way. SPACE is based on the CCM for psychosocial interventions, and core components were maintained to the fullest extent possible in the redesign. Patient-centered care was achieved with individualized teams composed of family, diabetes, and school supports. SPACE will allow for multiple referral reasons and sources, in line with population-based care. Measurement-based treatments focused on the evaluation of glycemia, quality of life, self-management skills, and time spent out of class for diabetes management. Finally, evidence-based care was translated to diabetes self-management and education practices.

SPACE is also distinctly unique from the original CCM in the extent to which it accommodates the differing environment (clinic vs school) and diagnosis (depression vs type 1 diabetes). Integrating a CCM into school poses new opportunities to reach a broader network of youth who may be underserved by the health care system. At the same time, there are inherent challenges to medical interventions in school. School health interventions need to consider the educational mission and pertinent outcomes, what medical services may or may not already be in place, and the different needs and wants of students and their parents [24]. Lyon et al [25] proposed key modifications to fit a CCM for the school setting for mental health care, including basing a care manager in school, allowing for flexible entry and treatments for a variety of mental health diagnoses, defining success both academically and medically, and incorporating school-wide supports. We carried these ideas forward to our school adaptation for type 1 diabetes.

Additional modifications to the intervention related to the more “physical” diagnosis of type 1 diabetes, rather than mental health care, though the framework for a school-based CCM nicely aligns with diabetes management. School nurses have frequent contact with these students for day-to-day and emergent care and can easily identify students who may benefit from this additional team support to help them achieve individualized goals [12]. Case management for youth with chronic disease is already considered one of the responsibilities of school nursing [47]. In some contrast to the original CCM, we planned for family engagement in SPACE. Parents may be highly involved in diabetes management both at home and at school, particularly for young children. We also allowed for flexible goal identification within SPACE, not solely focused on glycemia. Diabetes treatment is multifaceted, encompassing medication, nutrition, activity, and psychosocial aspects. This lends itself to measuring a variety of school and health-related outcomes to evaluate effectiveness. Our partners felt strongly that diabetes outcomes should include indicators of glycemia, self-management, quality of life, and academics.

The strengths of the SPACE model, identified by the design team and usability tests, focused on the core function of the multidisciplinary team. Having a common space for the student, school nurse, parent, and health care provider to meet was viewed as streamlining communication, giving personalized training to the school nurse, and building trust among all parties. The entirely technology-based SPACE intervention also heightened the perceived usability among school nurses and our design team. With increasing comfort with digital platforms generally, this condition was seen as more feasible for working parents and less intrusive to the school day. The design team had several unexpected suggestions. Some felt strongly the SPACE team should regularly include other school supports, including administrators, who may be less involved in day-to-day care. Others highlighted the role of school nurses to screen for social determinants of health that may influence diabetes management and offer universal resources to families to promote health equity. In usability testing, the SPACE model resonated with school nurses, who frequently described the activities as being within the scope of their role as a medical professional.

Despite the perceived advantages of the SPACE model, our usability testing did identify potential issues to be addressed prior to pilot-testing. The highest priority issues were feasible to address, including preparing school nurses and families to access the virtual platform and organizing secure tools for school nurses to share data with the research team. Other less pressing feedback related to intervention complexity includes coordinating a common time, engaging parents, and ensuring health office coverage. Solutions for these usability issues may need to be customized for different schools to carry out the core components of SPACE. Such an approach is acceptable and often necessary to promote the adoption and sustainability of complex interventions that are appropriately fit to the local context [46]. Though this may result in a tailoring of implementation strategies, adjusting features such as the virtual platform and processes for data collection will not alter the core functions of the intervention.

The iterative design cycles conducted over Zoom contributed to an efficient process, with all activities concluding within 6 months. We used several strategies to promote equitable cocreation practices and foster mutual trust and empathy among the community partners despite the web-based setting [48,49]. We demonstrated equity by including representation from different roles in the school and health system, as well as parents and individuals with type 1 diabetes, and compensating partners for their time and contributions. We addressed potential power imbalances by offering individual and group activities, including asking all parties to vote on ideas to limit the influence of any dominant voices. Applying a web-based format with two meeting options per month lowered barriers to participation like finding childcare, transportation costs, and time needed to participate. We emphasized reciprocity by summarizing and sharing back their comments and how these changed the prototype over time. We hoped to create a personalized and transformative experience by equipping them to participate in research in the future with research ethics training. Among the partners, many agreed to continue serving on a community advisory board, one school nurse volunteered her district to pilot-test SPACE, and a diabetes health care provider agreed to serve as an ongoing study consultant. Finally, we facilitated relationships by learning each other’s stories and personal motivations for joining this team.

### Limitations

The SPACE intervention was designed with community partners in a specific geographic area affiliated with our health system. Though this was intentional to ensure fit to our context, this may limit the generalizability of SPACE to other settings where there may be differences in school systems (eg, school health policy, ability to delegate insulin and glucagon, and school health staffing) or health systems (eg, size and resources of the diabetes center). We sought perspectives from nurses in different school districts to get broad representation from our region, though this is still reflective of Pennsylvania, specifically, and state laws may vary. Pennsylvania is one of 35 states with school nursing requirements, and like most states, the delegation of insulin and glucagon to trained lay staff is permitted. Depending on laws in other states, translation of SPACE may require an initial evaluation of the local policy, perceived barriers, and necessary modifications prior to implementation.

A second limitation relates to the composition of our design team. Though we included young adults with type 1 diabetes, we did not rigorously incorporate perspectives of youth with diabetes. Our parent participants did informally discuss the intervention with their children between design meetings, which they shared with us. The SPACE intervention will be piloted with elementary and middle school–aged children (12 years of age or younger), and children in this age group may not have been able to participate in our activities as designed. Older teens were not included as developmentally, their priorities for diabetes management in school will differ from those of younger children who are more reliant on their parents and school nurses.

Finally, the identification of usability issues is inherently a subjective process. It is possible that additional usability issues will arise in future testing. However, a strength of this study was the inclusion of school nurses and other roles from different school districts in our state.

### Comparison With Prior Work

SPACE represents a shift from other school-based diabetes interventions by integrating school nurses into the diabetes medical team with parental support and establishing continuity in that relationship. Prior interventions have focused on school nurses alone, including delivering educational tools and curricula, case management, or engaging school nurses to deliver some diabetes tasks (downloading devices and giving long-acting insulin) [50-53]. Other interventions offer visits from diabetes providers in the school setting, such as self-management education and telemedicine [54,55]. Generally, these interventions have improved school nurse’s knowledge of diabetes, and some have demonstrated a small improvement in hemoglobin A_1c_ [53,55]. To date, there is limited data on the sustainability or impact of prior interventions, and none are endorsed by leading diabetes organizations as best practices. In contrast, school-based asthma interventions are better studied. Asthma interventions that emphasize care coordination and parent engagement have demonstrated a reduction in hospital admissions and improvements in asthma-related quality of life [56]. A core pillar of the Centers for Disease Control and Prevention–sponsored asthma-friendly schools program is the coordination of school, family, and community efforts to better manage symptoms and reduce absenteeism. The use of UCD methods for co-design, paired with the USE-EBPI, will hopefully enhance the potential reach and impact of SPACE in future testing.

### Conclusions

We present the iterative cocreation of SPACE, a multidisciplinary, goal-directed, school-partnered diabetes intervention based on the evidenced-based CCM for depression management. Relying on UCD methodology, we involved diverse community partners at all phases of the intervention design with the consolidation of ideas on a final prototype that is ready for formal testing. Our use of videoconferencing and shared digital whiteboards enabled diverse participation in a relatively short time interval. The USE-EBPI methods for usability testing helped to evaluate the quality of our design process, establishing a bridge between UCD and IS research. The quantitative indicators suggested a high degree of usability among school nurses of different backgrounds, which was reflected in their comments about how they would operationalize SPACE in their school district. Though cross-sector interventions are by their nature complex, this staged approach to intervention adaptation and preliminary testing may help to overcome barriers and establish a strong foundation for future implementation.

## Appendix

Multimedia Appendix 1 Images representing Mural design activities in session/cycle 1. The first image displays a sample creative matrix with design ideas; the second image adds the priority ranking, represented by the red and blue dots to indicate first and second priority, respectively.

Multimedia Appendix 2 Low-fidelity prototype (concept poster) of the SPACE intervention for critique in session/cycle 2. SPACE: school-partnered collaborative care.

Multimedia Appendix 3 Narrative storyboard of the "SPACE for type 1 diabetes" prototype used for cognitive walkthroughs to assess usability with school nurses. SPACE: school-partnered collaborative care.

## Abbreviations

CCM: collaborative care model
IS: implementation science
IUS: intervention usability scale
SPACE: school-partnered collaborative care
UCD: user-centered design

## Data Availability

The datasets generated or analyzed during this study are available from the corresponding author upon reasonable request.

## References

[1] Helgeson VS, Lopez LC, Kamarck T (2009). Peer relationships and diabetes: retrospective and ecological momentary assessment approaches. Health Psychol.

[2] Helgeson VS, Palladino DK, Reynolds KA, Becker D, Escobar O, Siminerio L (2014). Early adolescent relationship predictors of emerging adult outcomes: youth with and without type 1 diabetes. Ann Behav Med.

[3] Helgeson VS, Horner FS, Reis HT, Niezink NMD, Libman I (2023). Support and conflict among youth with type 1 diabetes: a focus on friends. J Pediatr Psychol.

[4] Raymaekers K, Oris L, Prikken S, Moons P, Goossens E, Weets I, Luyckx K (2017). The role of peers for diabetes management in adolescents and emerging adults with type 1 diabetes: a longitudinal study. Diabetes Care.

[5] Walker AF, Addala A, Sheehan E, Lal R, Haller M, Cuttriss N, Filipp S, Baer L, Gurka M, Bernier A, Figg L, Westen S, Hood K, Anez-Zabala C, Frank E, Roque X, Maizel J, Maahs D (2022). Using peer power to reduce health disparities: implementation of a diabetes support coach program in federally qualified health centers. Diabetes Spectr.

[6] Hilliard ME, Tully C, Monaghan M, Hildebrandt T, Wang CH, Barber JR, Clary L, Gallagher K, Levy W, Cogen F, Henderson C, Karaviti L, Streisand R (2022). First STEPS: primary outcomes of a randomized, stepped-care behavioral clinical trial for parents of young children with new-onset type 1 diabetes. Diabetes Care.

[7] Tully C, Shneider C, Monaghan M, Hilliard ME, Streisand R (2017). Peer coaching interventions for parents of children with type 1 diabetes. Curr Diab Rep.

[8] Ross CE, Wu C (1995). The links between education and health. Am Sociol Rev.

[9] March CA, Leikam L, Siminerio LM, Miller E, Libman IM (2021). Cyber school is a marker of youth with high-risk diabetes. J Pediatr.

[10] March CA, Siminerio LM, Muzumdar RH, Libman IM (2021). Implications of the school day on health behaviors for children with type 1 diabetes: a survey of parent perspectives during the COVID-19 pandemic. Sci Diabetes Self Manag Care.

[11] Cogen F, Rodriguez H, March CA, Muñoz CE, McManemin J, Pellizzari M, Rodriguez J, Wyckoff L, Yatvin AL, Atkinson T, ElSayed NA, Bannuru RR, Pekas EJ, Woodward C, Sherman J (2024). Diabetes care in the school setting: a statement of the American diabetes association. Diabetes Care.

[12] March CA, Nanni M, Kazmerski TM, Siminerio LM, Miller E, Libman IM (2020). Modern diabetes devices in the school setting: perspectives from school nurses. Pediatr Diabetes.

[13] Kobos E, Imiela J, Kryczka T, Szewczyk A, Knoff B (2020). Actual and perceived knowledge of type 1 diabetes mellitus among school nurses. Nurse Educ Today.

[14] Puckett C, Wong JC, Talbot S, Min HJ, Chokr N (2023). Institutional role conflict in the digital age: the case of diabetes management at school. SSM Qual Res Health.

[15] Uhm JY, Choi MY (2020). Barriers to and facilitators of school health care for students with chronic disease as perceived by their parents: a mixed systematic review. Healthcare (Basel).

[16] Kise SS, Hopkins A, Burke S (2017). Improving school experiences for adolescents with type 1 diabetes. J Sch Health.

[17] March CA, Nanni M, Lutz J, Kavanaugh M, Jeong K, Siminerio LM, Rothenberger S, Miller E, Libman IM (2023). Comparisons of school-day glycemia in different settings for children with type 1 diabetes using continuous glucose monitoring. Pediatr Diabetes.

[18] March CA, Siminerio LM, Kazmerski TM, Albanese-O'Neill A, Miller E, Libman I (2023). School-based diabetes care: a national survey of U.S. pediatric diabetes providers. Pediatr Diabetes.

[19] Pansier B, Schulz PJ (2015). School-based diabetes interventions and their outcomes: a systematic literature review. J Public Health Res.

[20] An R, Li D, Cole M, Park K, Lyon AR, White NH (2022). Implementation of school diabetes care in the United States: a scoping review. J Sch Nurs.

[21] Asarnow JR, Rozenman M, Wiblin J, Zeltzer L (2015). Integrated medical-behavioral care compared with usual primary care for child and adolescent behavioral health: a meta-analysis. JAMA Pediatr.

[22] Richardson LP, Ludman E, McCauley E, Lindenbaum J, Larison C, Zhou C, Clarke G, Brent D, Katon W (2014). Collaborative care for adolescents with depression in primary care: a randomized clinical trial. JAMA.

[23] Katon WJ, Lin EHB, Von Korff M, Ciechanowski P, Ludman EJ, Young B, Peterson D, Rutter CM, McGregor M, McCulloch D (2010). Collaborative care for patients with depression and chronic illnesses. N Engl J Med.

[24] Lyon AR, Whitaker K, French WP, Richardson LP, Wasse JK, McCauley E (2016). Collaborative care in schools: enhancing integration and impact in youth mental health. Adv Sch Ment Health Promot.

[25] Lyon AR, Whitaker K, Richardson LP, French WP, McCauley E (2019). Collaborative care to improve access and quality in school-based behavioral health. J Sch Health.

[26] Melles M, Albayrak A, Goossens R (2021). Innovating health care: key characteristics of human-centered design. Int J Qual Health Care.

[27] Chen E, Neta G, Roberts MC (2021). Complementary approaches to problem solving in healthcare and public health: implementation science and human-centered design. Transl Behav Med.

[28] Lyon AR, Koerner K (2016). User-centered design for psychosocial intervention development and implementation. Clin Psychol (New York).

[29] Wheelock A, Bechtel C, Leff B (2020). Human-centered design and trust in medicine. JAMA.

[30] Munson SA, Friedman EC, Osterhage K, Allred R, Pullmann MD, Areán PA, Lyon AR (2022). Usability issues in evidence-based psychosocial interventions and implementation strategies: cross-project analysis. J Med Internet Res.

[31] Lyon AR, Brewer SK, Areán PA (2020). Leveraging human-centered design to implement modern psychological science: return on an early investment. Am Psychol.

[32] Dopp AR, Parisi KE, Munson SA, Lyon AR (2019). A glossary of user-centered design strategies for implementation experts. Transl Behav Med.

[33] Wiltsey Stirman S, Baumann AA, Miller CJ (2019). The FRAME: an expanded framework for reporting adaptations and modifications to evidence-based interventions. Implement Sci.

[34] March CA, Kazmerski TM, Moon C, Libman IM, Miller E (2021). Evaluating the impact of stakeholder engagement in a school-based type 1 diabetes study. Diabetes Spectr.

[35] Saunders T, Mackie TI, Shah S, Gooding H, de Ferranti SD, Leslie LK (2016). Young adult and parent stakeholder perspectives on participation in patient-centered comparative effectiveness research. J Comp Eff Res.

[36] Yonas MA, Jaime MC, Barone J, Valenti S, Documét P, Ryan CM, Miller E (2016). Community partnered research ethics training in practice: a collaborative approach to certification. J Empir Res Hum Res Ethics.

[37] Witteman HO, Vaisson G, Provencher T, Chipenda Dansokho S, Colquhoun H, Dugas M, Fagerlin A, Giguere AM, Haslett L, Hoffman A, Ivers NM, Légaré F, Trottier M, Stacey D, Volk RJ, Renaud J (2021). An 11-item measure of user- and human-centered design for personal health tools (UCD-11): development and validation. J Med Internet Res.

[38] Coulter RWS, Gartner RE (2023). LGBTQ+ youth-generated intervention concepts for reducing teen dating violence inequities. Health Promot Pract.

[39] Lyon AR, Koerner K, Chung J (2020). Usability evaluation for evidence-based psychosocial interventions (USE-EBPI): a methodology for assessing complex intervention implementability. Implement Res Pract.

[40] Nielsen J, Landauer TK (1993). A mathematical model of the finding of usability problems.

[41] Faulkner L (2003). Beyond the five-user assumption: benefits of increased sample sizes in usability testing. Behav Res Methods Instrum Comput.

[42] Lyon AR, Pullmann MD, Jacobson J, Osterhage K, Achkar MA, Renn BN, Munson SA, Areán PA (2021). Assessing the usability of complex psychosocial interventions: the intervention usability scale. Implement Res Pract.

[43] Bangor A, Kortum PT, Miller JT (2008). An empirical evaluation of the system usability scale. Int J Hum-Comput Interact.

[44] March CA, Hill A, Kazmerski TM, Siminerio L, Switzer G, Miller E, Libman I (2023). School nurse confidence with diabetes devices in relation to diabetes knowledge and prior training: a study of convergent validity. Pediatr Diabetes.

[45] Herlitz L, MacIntyre H, Osborn T, Bonell C (2020). The sustainability of public health interventions in schools: a systematic review. Implement Sci.

[46] Perez Jolles M, Lengnick-Hall R, Mittman BS (2019). Core functions and forms of complex health interventions: a patient-centered medical home illustration. J Gen Intern Med.

[47] Council On School Health (2016). Role of the school nurse in providing school health services. Pediatrics.

[48] Pérez Jolles M, Willging CE, Stadnick NA, Crable EL, Lengnick-Hall R, Hawkins J, Aarons GA (2022). Understanding implementation research collaborations from a co-creation lens: recommendations for a path forward. Front Health Serv.

[49] Bazzano AN, Noel LA, Patel T, Dominique CC, Haywood C, Moore S, Mantsios A, Davis PA (2023). Improving the engagement of underrepresented people in health research through equity-centered design thinking: qualitative study and process evaluation for the development of the grounding health research in design toolkit. JMIR Form Res.

[50] Pollock AJ, Beaton WN, Burgess BA, Logel SN, Wilson L, Ciha JE, Allen J (2023). Diabetes in school health (DiSH): telementoring collaboration between pediatric diabetes specialists and school nurses to improve care of children with diabetes. J Sch Nurs.

[51] Peery AI, Engelke MK, Swanson MS (2012). Parent and teacher perceptions of the impact of school nurse interventions on children's self-management of diabetes. J Sch Nurs.

[52] Tonyushkina KN, Cobb V, Lawson G, Dunn K, Pelzek J, Blain T, Clancy J, Allen H (2021). Challenges and opportunities of engaging school nurses in a diabetes care team in a culturally diverse lowincome community—a mixed-methods feasibility study. Health Behav Policy Rev.

[53] Nguyen TM, Mason KJ, Sanders CG, Yazdani P, Heptulla RA (2008). Targeting blood glucose management in school improves glycemic control in children with poorly controlled type 1 diabetes mellitus. J Pediatr.

[54] Faro B, Ingersoll G, Fiore H, Ippolito KS (2005). Improving students' diabetes management through school-based diabetes care. J Pediatr Health Care.

[55] Izquierdo R, Morin PC, Bratt K, Moreau Z, Meyer S, Ploutz-Snyder R, Wade M, Weinstock RS (2009). School-centered telemedicine for children with type 1 diabetes mellitus. J Pediatr.

[56] Harris K, Kneale D, Lasserson TJ, McDonald VM, Grigg J, Thomas J (2019). School-based self-management interventions for asthma in children and adolescents: a mixed methods systematic review. Cochrane Database Syst Rev.

