# Supplementary figures and images for: School-Partnered Collaborative Care (SPACE) for Pediatric Type 1 Diabetes: Development and Usability Study of a Virtual Intervention With Multisystem Community Partners
Source: JMIR Diabetes. 2025 Mar 26;10:e64096. doi: 10.2196/64096 (PMC11982762; doi:10.2196/64096)

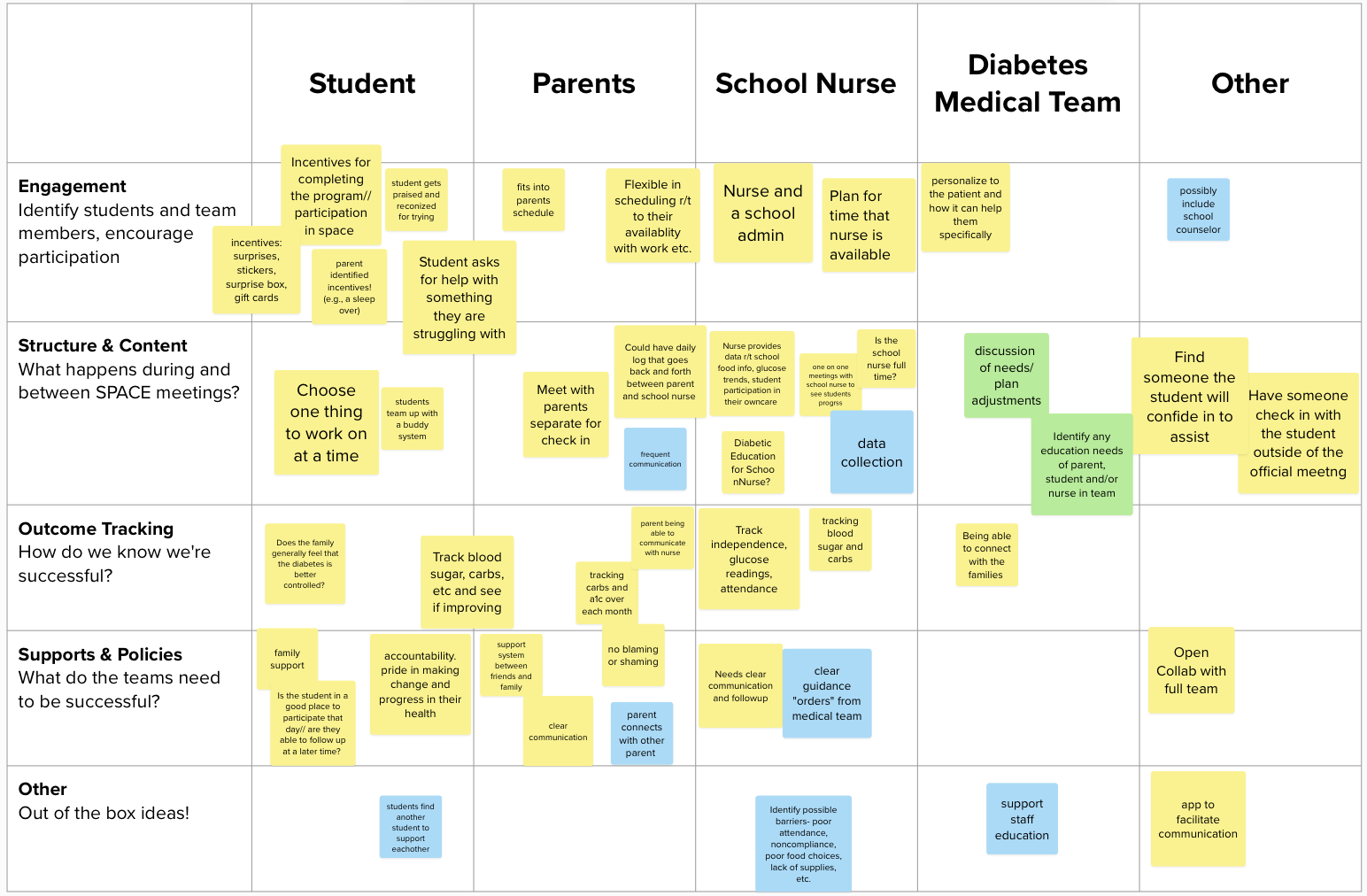

Supplement: Multimedia Appendix 1 [file diabetes_v10i1e64096_app1.png]
