# Supplementary material for: School-Partnered Collaborative Care (SPACE) for Pediatric Type 1 Diabetes: Development and Usability Study of a Virtual Intervention With Multisystem Community Partners
Source: JMIR Diabetes. 2025 Mar 26;10:e64096. doi: 10.2196/64096 (PMC11982762; doi:10.2196/64096)
Supplement: Multimedia Appendix 2 [file diabetes_v10i1e64096_app2.pdf]

School Partnered Collaborative Care

SPACE

FOR TYPE 1 DIABETES

Target Population*

any student with T1D

≥ 2 emergency room visits or admissions in the past year

missing 2 or more visits with diabetes provider

>7%

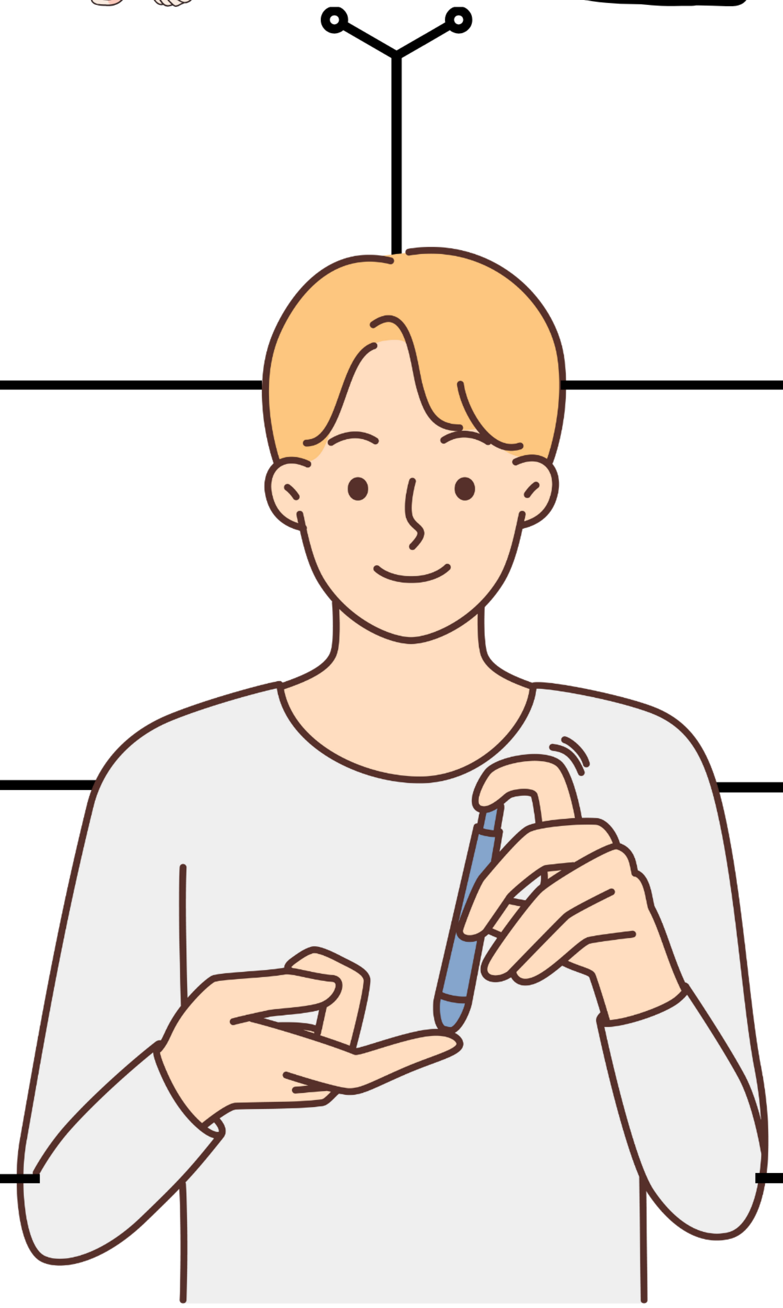

limited support from peers/family

frequent ketones/hypoglycemia at school

not self-managing

distress with diabetes management

*in this pilot, we will not target specific kids, but for future studies, children can be **referred** to the program by self/parent/school nurse/medical team for the above criteria*

Structure

30 min meetings

Virtual Platform

Occur Monthly

Multiple students can be batched on the same day if better for finding coverage for the health office

Scheduled by research team at a time that works for school nurse and the parent

Can be scheduled immediately before or after school or during school day

Mandatory attendees: parent/guardian, student, school nurse, diabetes care and education specialist

Optional attendees: school admin, school counselor, school social worker

Parent will give permission for school nurse to meet with DCES even if parent cannot be present

Meeting Members

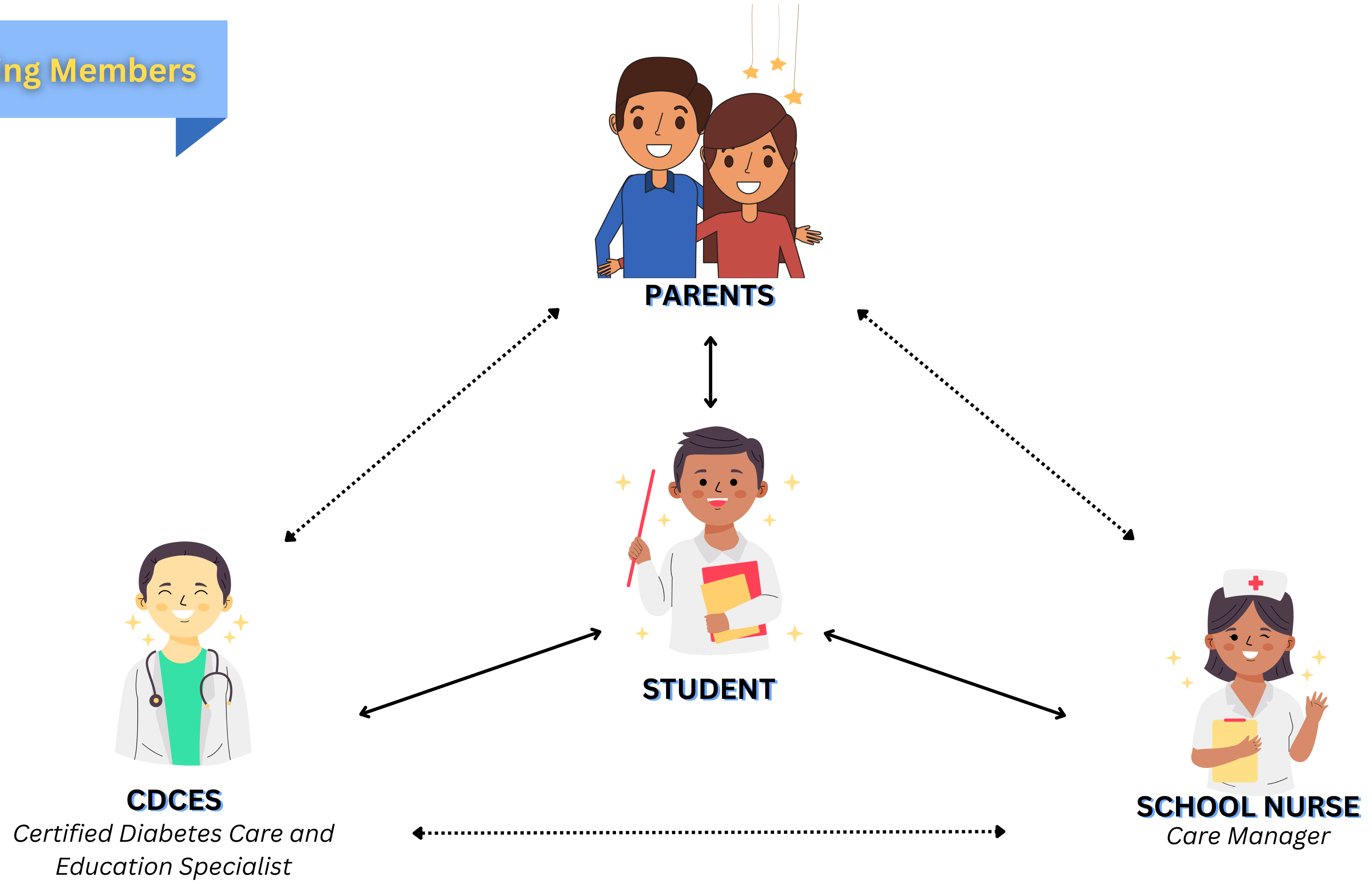

Content of Meetings

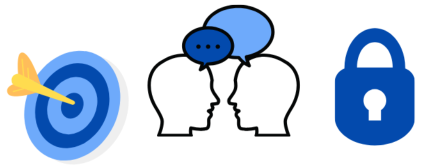

STARTS WITH DISCLAIMER

- *Goals of why we are here*
- *Need to use common and respectful language*
- *Will respect privacy of the student*
- *Focus on opportunities*

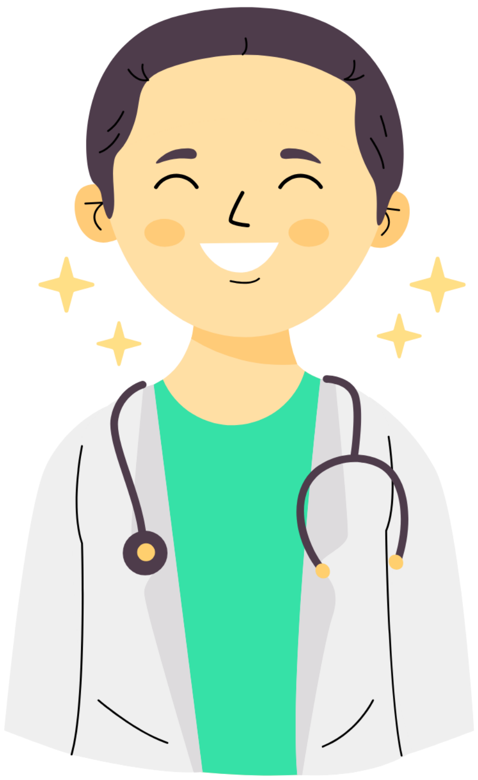

CDCES

- Make changes to IHP/school orders
- Follow a template for meeting notes
- Notes sent to all team members and provider
- Provide educational resources to school nurse as needed

Content of Meetings

- Goal identification
- Assess barriers/progress
- Discussion solutions
  - Review glucose data
  - Counseling/education
  - School or health-related referrals
- Select 1-3 tangible things to work on/change
- Create a plan for home and school

Examples of referrals

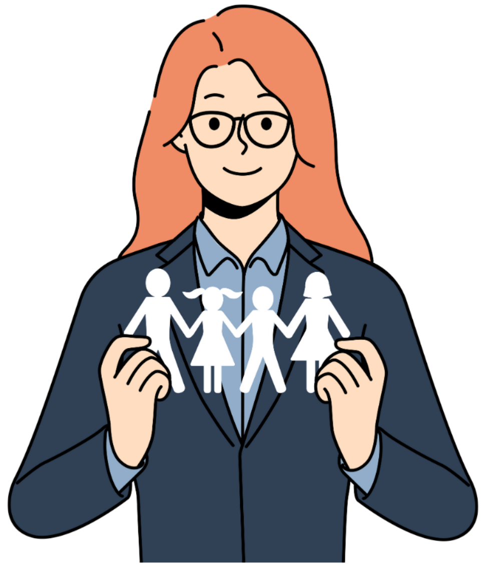

Social Work

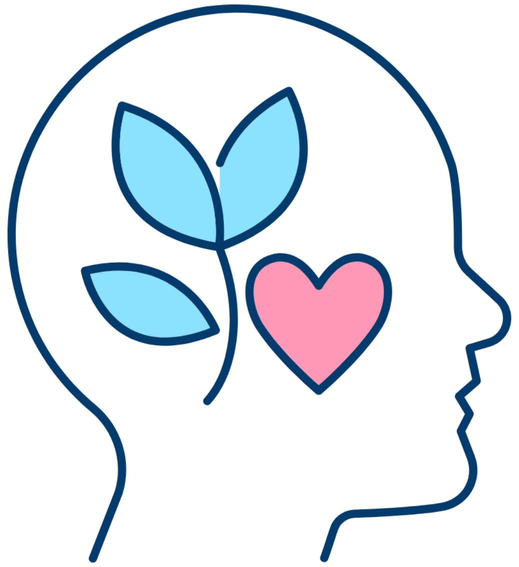

Behavioral Health

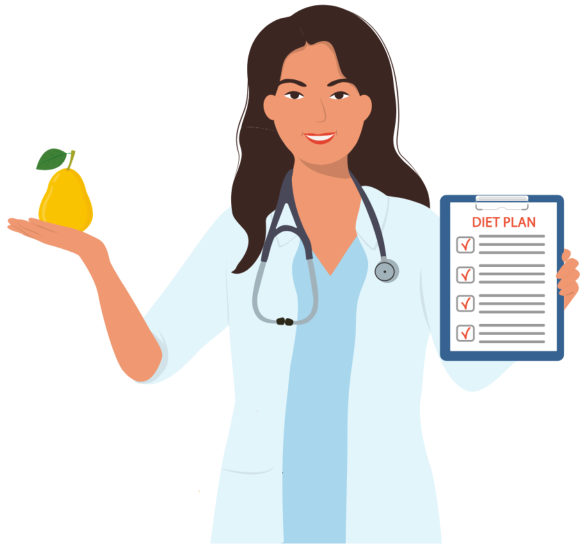

Dietician

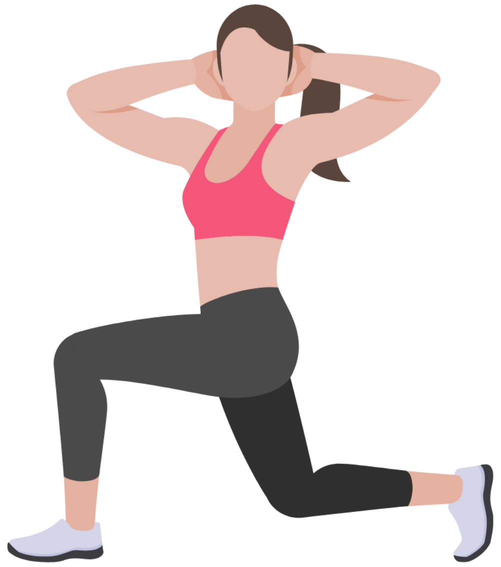

Gyms/YMCAs

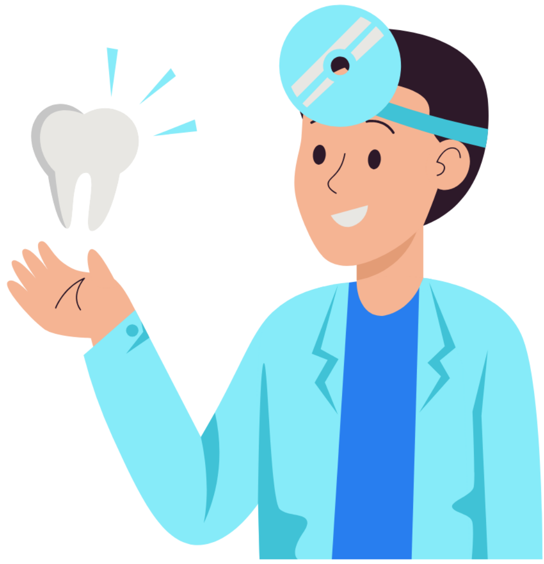

Dentist

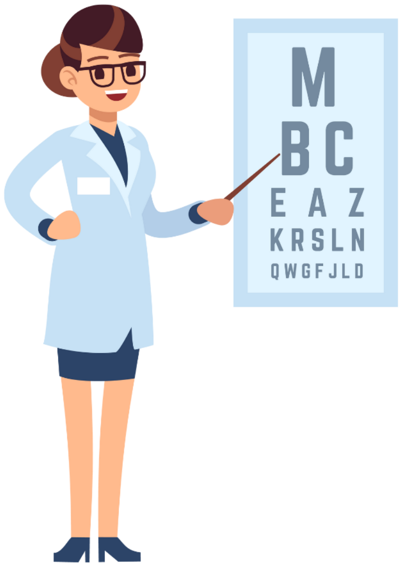

Ophthalmology

(if > 11 years old)

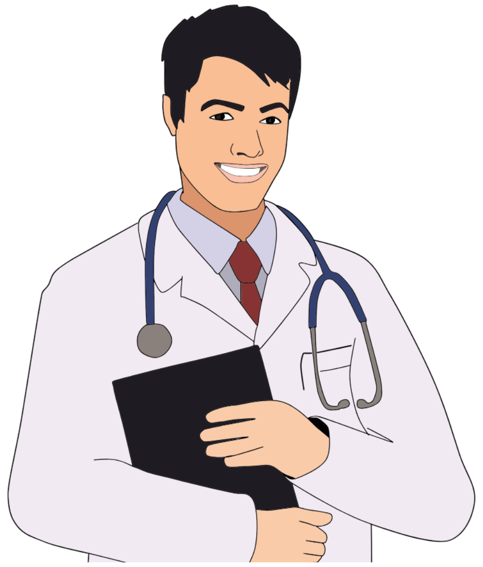

Primary Care

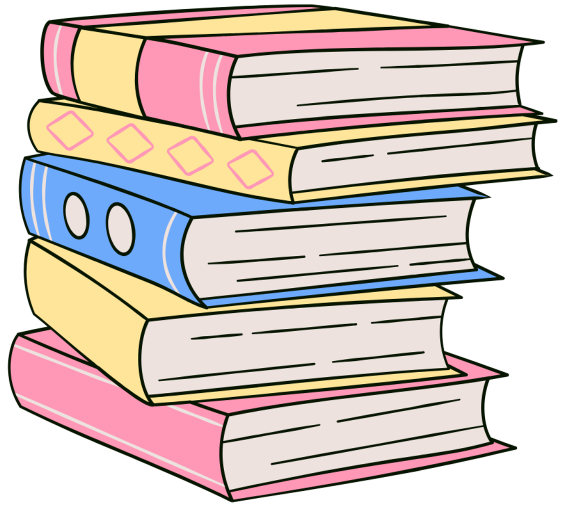

Academic Support

(504/IEP teams, student assistance team, school counselor)

Between Meetings at home

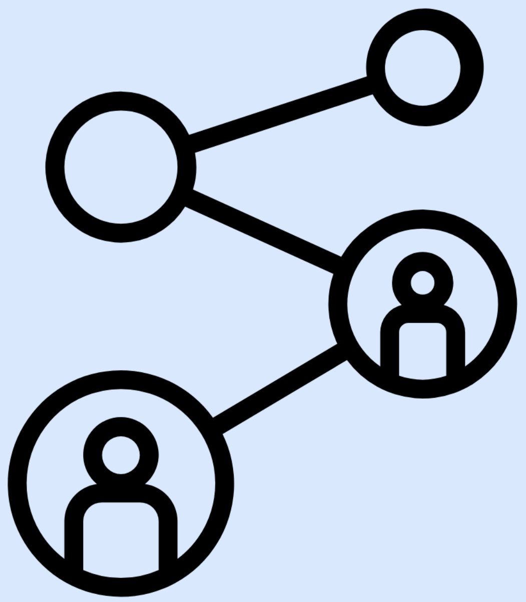

Parents follow up with referrals

Helps student with pre-specified goals and tracks progress

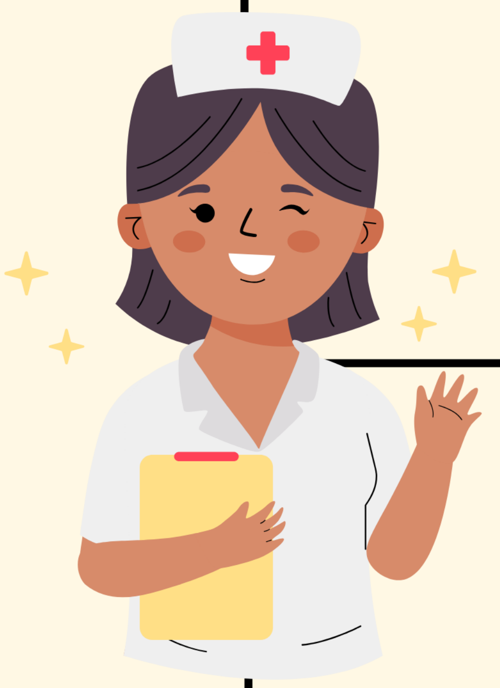

Communicate with parents at least once weekly

Can call diabetes center if needed/ urgent

Between Meetings at school

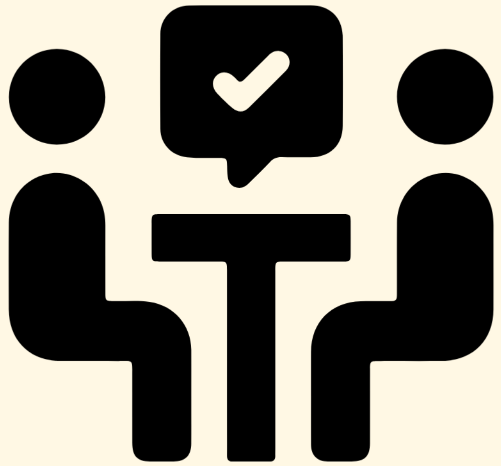

Team member (nurse/counselor/admin) checks in with student

Outcomes to track clinically

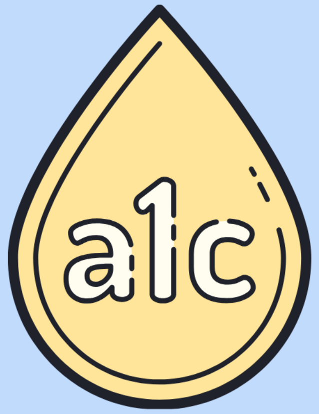

at clinic visits

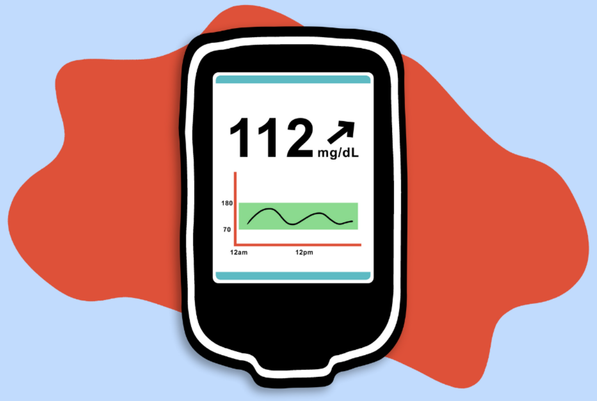

monthly CGM/glucometer data

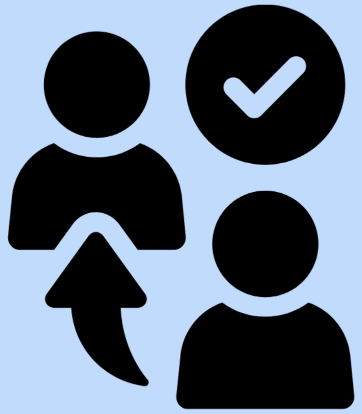

referrals made/scheduled/completed

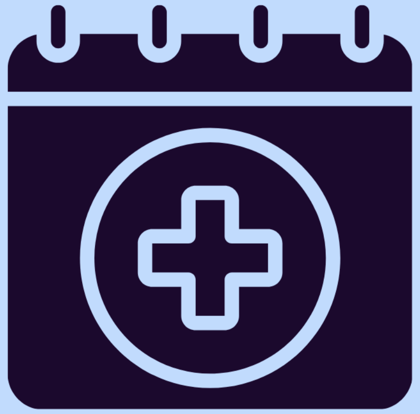

appointment attendance

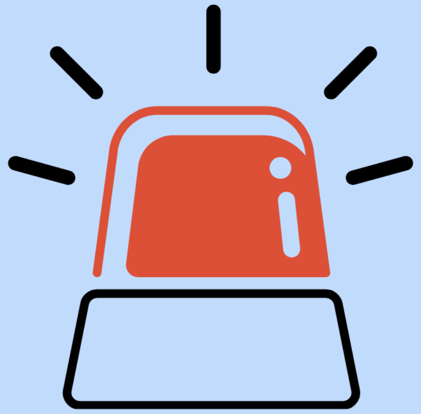

diabetes related ER/admissions

Outcomes to track at School

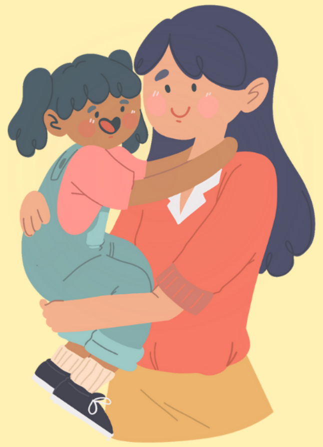

diabetes quality of life/distress

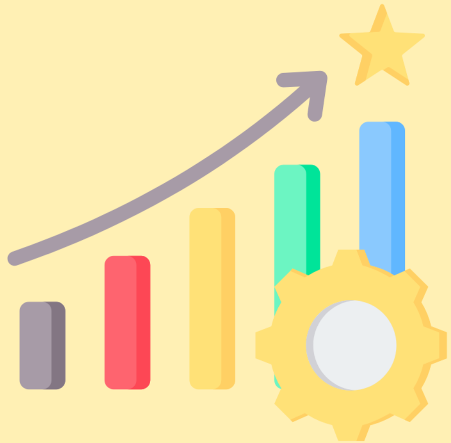

capability for self-management

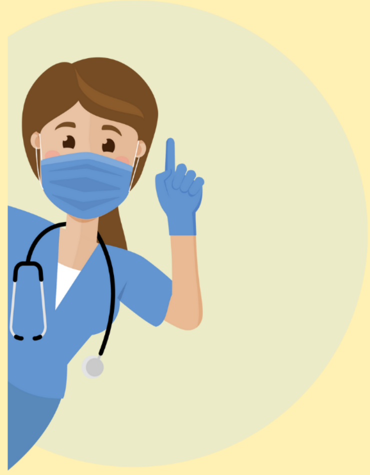

unscheduled school nurse visits and related communication

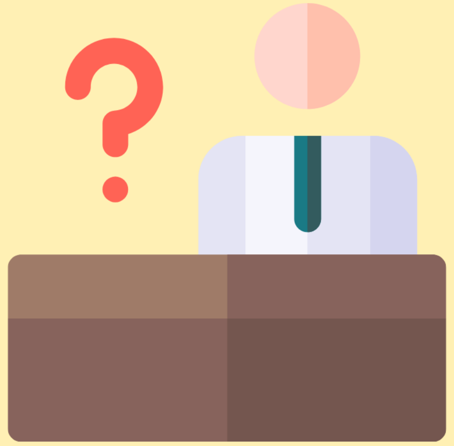

diabetes related absences/tardines/ dismissals

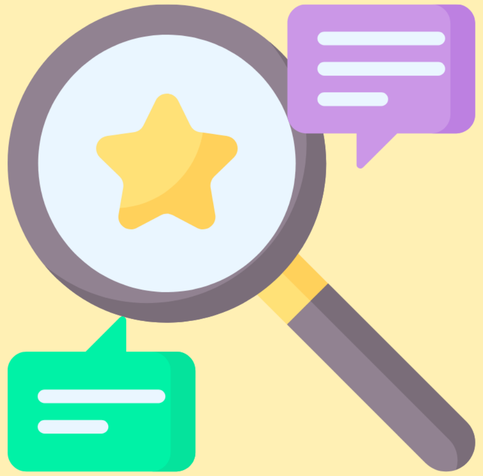

personal experience in SPACE
