# Supplementary material for: School-Partnered Collaborative Care (SPACE) for Pediatric Type 1 Diabetes: Development and Usability Study of a Virtual Intervention With Multisystem Community Partners
Source: JMIR Diabetes. 2025 Mar 26;10:e64096. doi: 10.2196/64096 (PMC11982762; doi:10.2196/64096)
Supplement: Multimedia Appendix 3 [file diabetes_v10i1e64096_app3.pdf]

School-Partnered Collaborative carE(SPACE)

For pediatric type 1 diabetes

Today's assignment

Your school district has agreed to participate in a new diabetes intervention. We are going to walk through each step of the intervention and get your feedback. The intervention is called school-partnered collaborative care, which we are abbreviating SPACE, for pediatric type 1 diabetes. This involves collaborative meetings between a school nurse, student, their parent/guardian, and a member of their diabetes medical team. When needed, other school support might join the meeting. We will have you go through each step and **talk aloud** your thoughts as you go.

Today’s assignment

On each slide, you can talk aloud about what you see and what you think about it.

We invite you to consider:

- *What’s happening here?*
- *When would this happen?*
- *How would this happen?*
- *What do you think about it?*

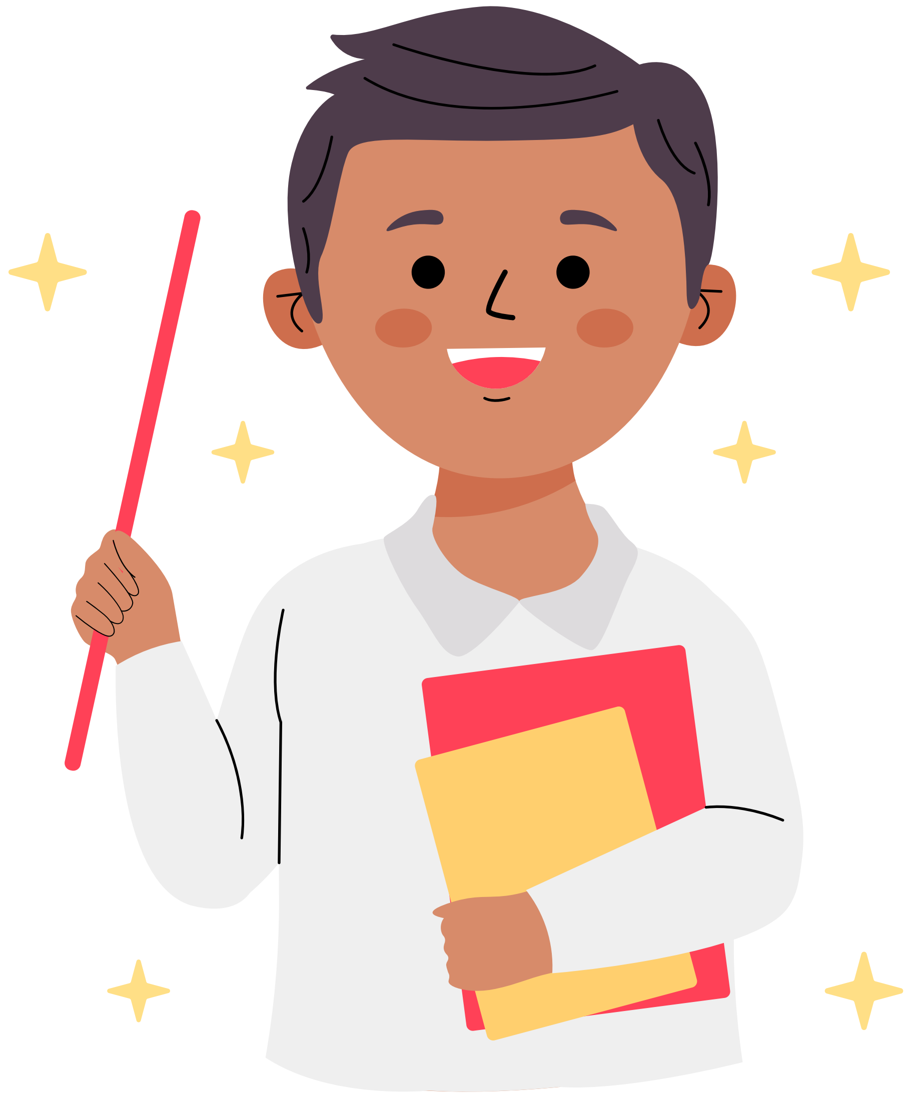

STUDENT

This is Tyler. He is 8 years old. He has had type 1 diabetes for two years, and he uses pens. His HbA1c is 9.0%, and he is not yet giving his own insulin, though he can check a blood sugar.

You notice he has a lot of high blood sugars at school. You think he might be a good candidate for SPACE and decide to approach his parents.

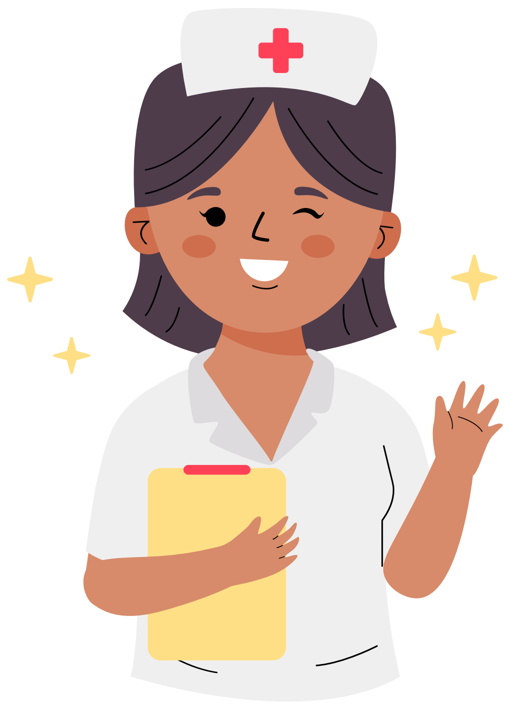

SCHOOL NURSE

I noticed Tyler is having some high blood sugars. We are partnering with Children's Hospital in a new program, called SPACE.

We'll all meet regularly with his diabetes medical team to help Tyler work on specific diabetes goals. Children's will ask your permission to participate!

Sounds great!

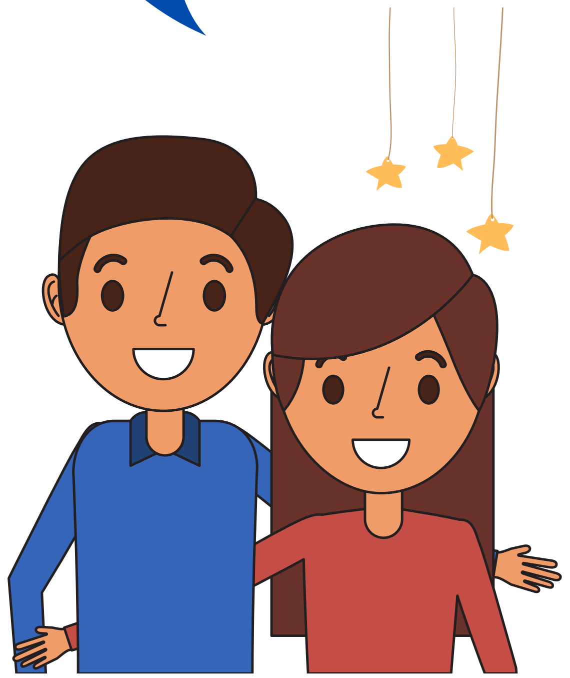

PARENTS

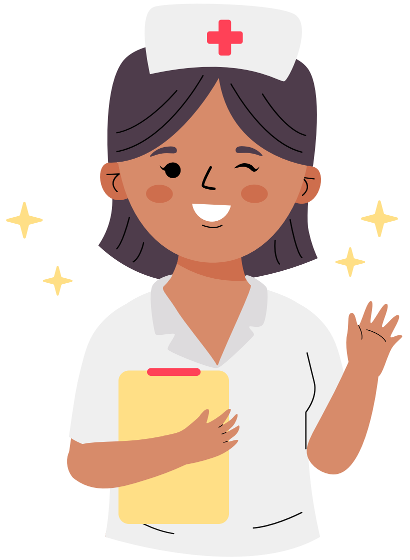

SCHOOL NURSE

First, we convene the team.
This team has a common goal: to help Tyler. Let's meet everyone!

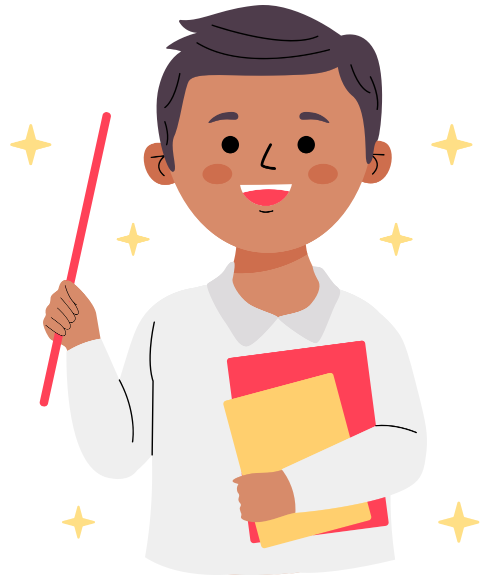

STUDENT

Tyler's parent/ guardian

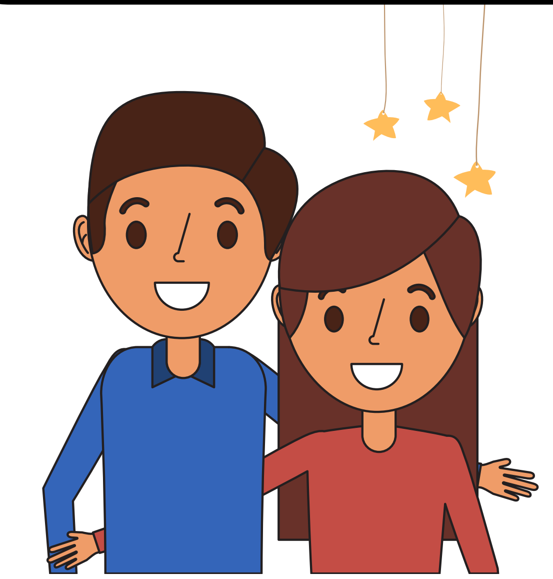

PARENTS

Our expert consultant

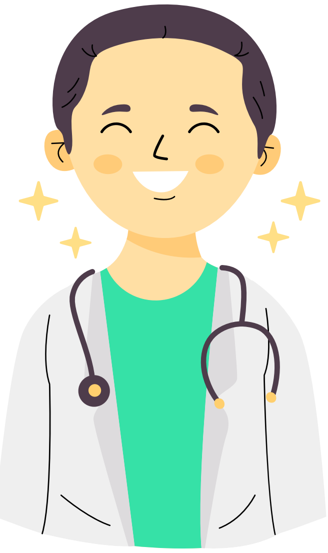

DIABETES MEDICAL TEAM

Other optional school support

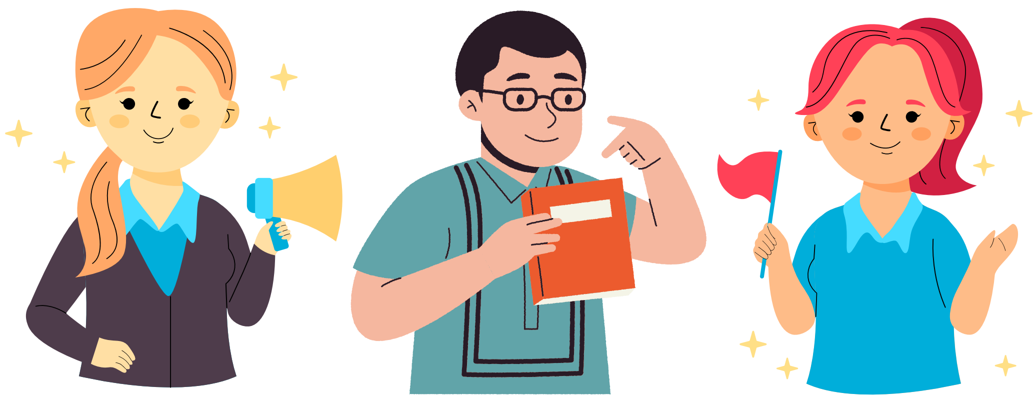

SCHOOL SUPPORT

Administrator, teachers, guidance counselor, behavioral therapist, coach, etc.

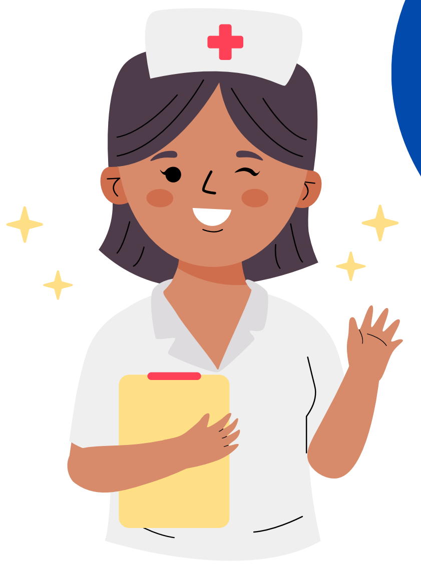

SCHOOL NURSE

I'll be the care manager, as I see Tyler every day at school. The Medical Team expert will be the Certified Diabetes Care and Education Specialist. We always have the option of adding optional school support when we feel a need.
It looks something like this:

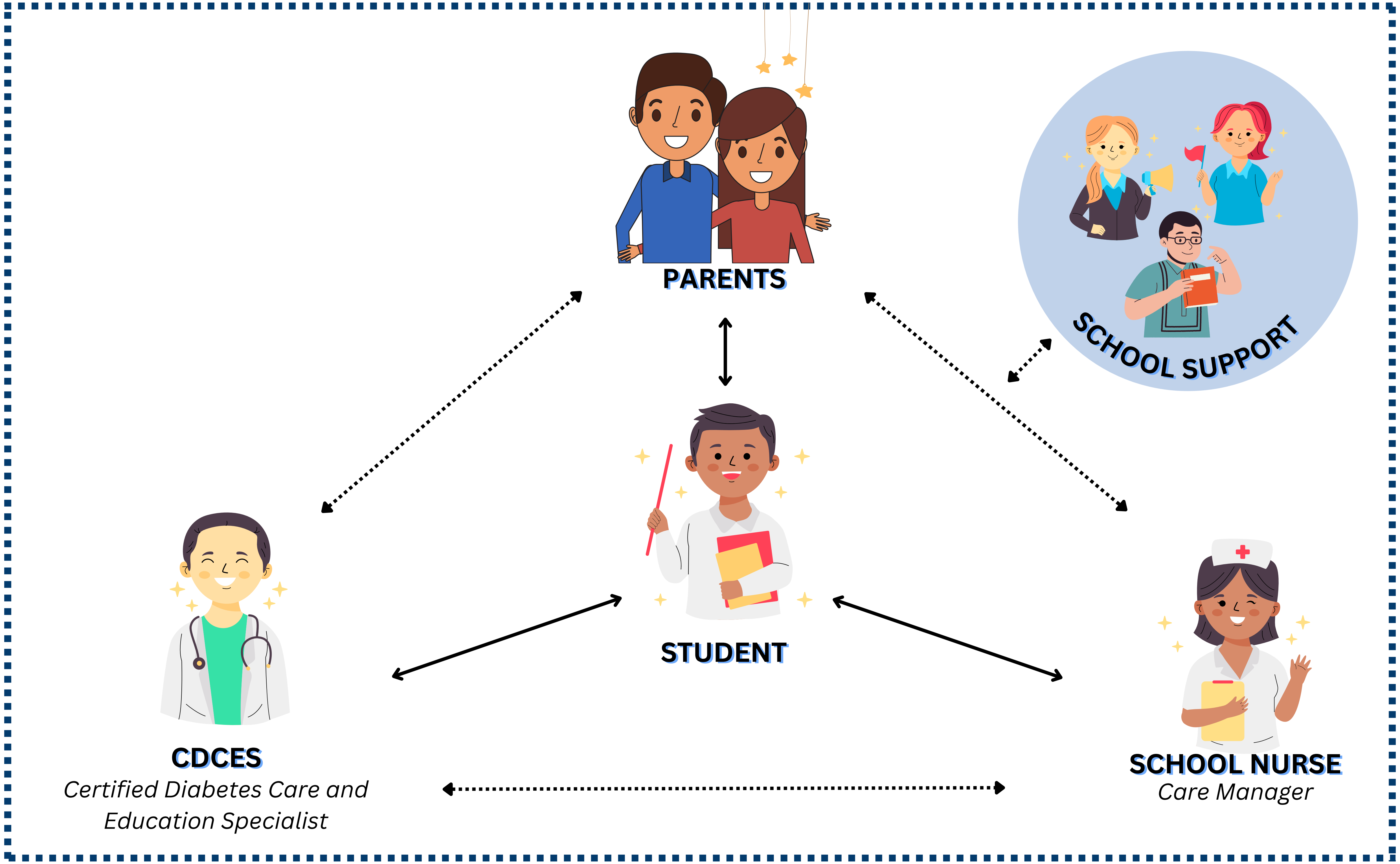

Our first meeting will be in a few weeks. It will be **30 minutes** and **virtual**, so you can join from your home/work.

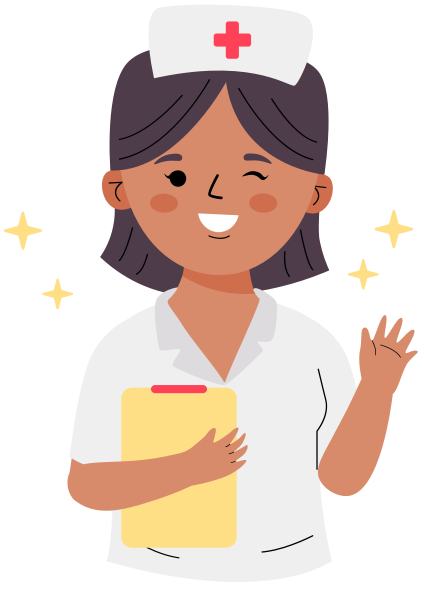

SCHOOL NURSE

What happens if we can't make a future meeting?

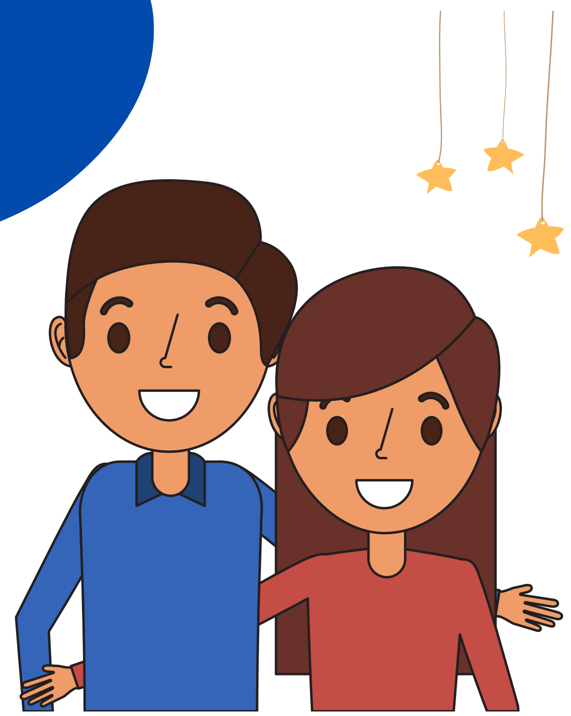

PARENTS

Our hope is that you can come to the majority of the meetings, but we know that something may come up. In that case, we will ask your permission to meet and then follow up with you.

I will first look at my availability and try to propose timings when I'm not that busy. I will do my best to make sure my health office is covered at that time.

Tyler will be excused from class, ideally when he has a more flexible period.

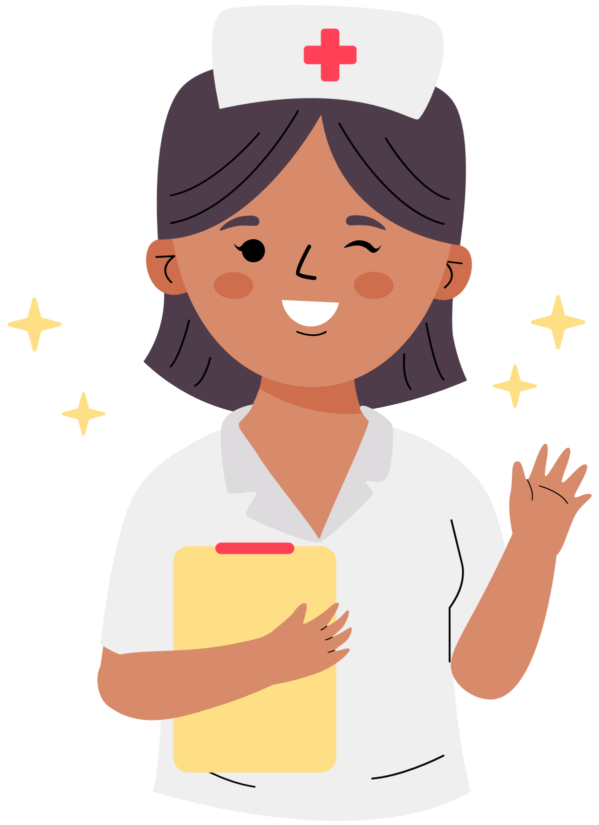

SCHOOL NURSE

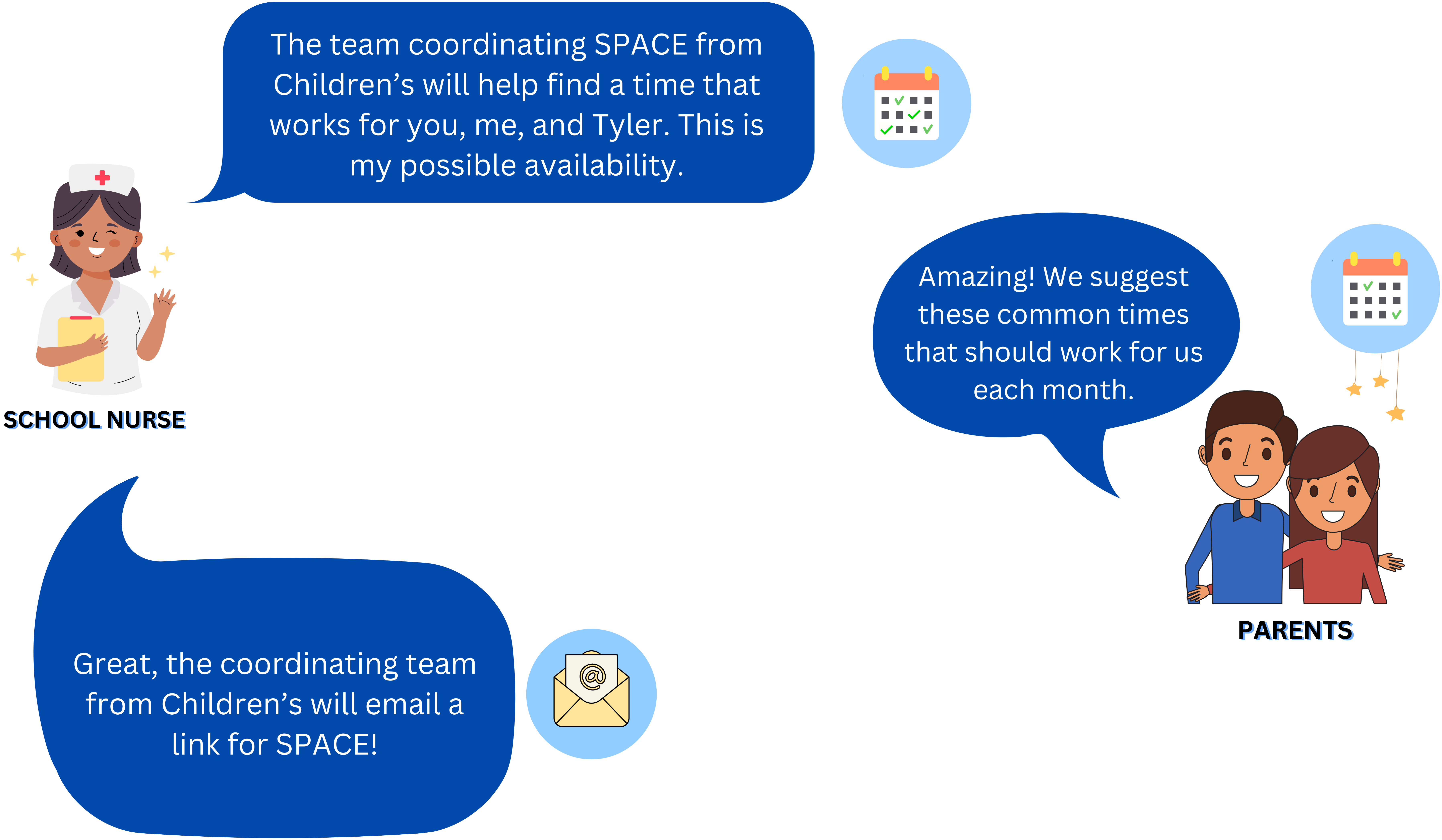
The team coordinating SPACE from Children's will help find a time that works for you, me, and Tyler. This is my possible availability.
SCHOOL NURSE
Amazing! We suggest these common times that should work for us each month.
PARENTS
Great, the coordinating team from Children's will email a link for SPACE!

Before our first meeting, think about what you hope to get out of SPACE.

Tyler’s blood sugars have been high. We think he is sneaking food. He has been wanting to sleep-over at his friend’s place for a while but we were too worried about his numbers to allow it. Children's has seen this on his CGM.

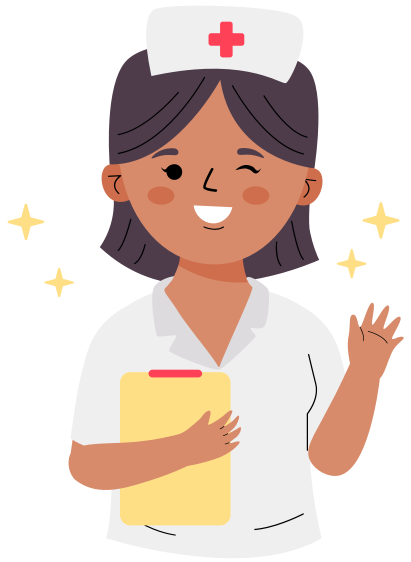

SCHOOL NURSE

I hate feeling different from my friends.
I’m scared to give shots.
Sometimes I sneak food.
I want to join my friends for sleepovers.

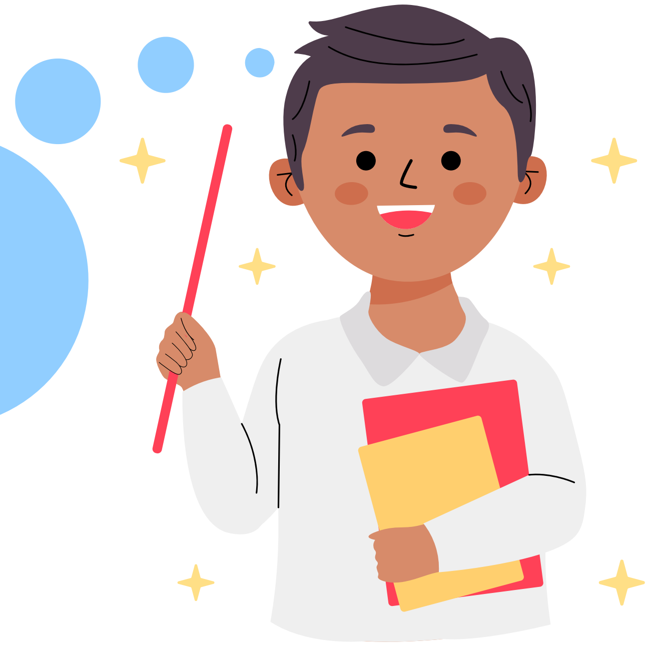

STUDENT

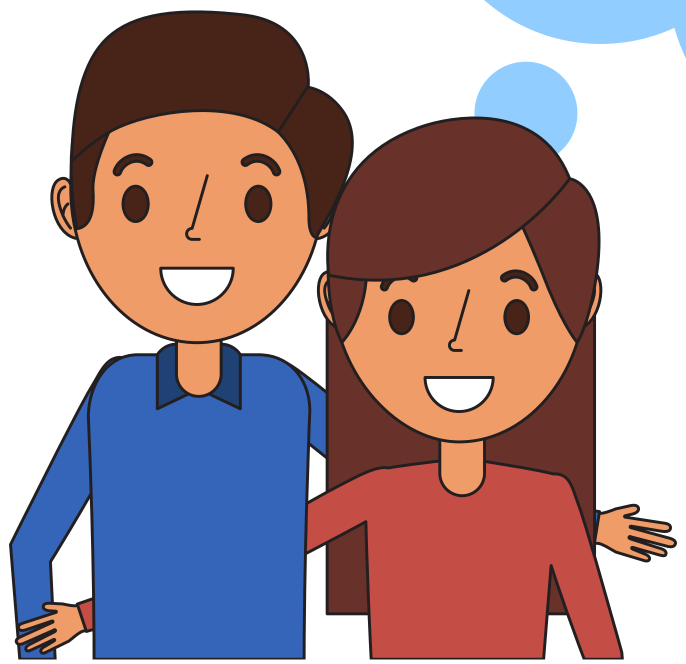

PARENTS

First Meeting

Hi everyone. Thanks for logging on. Before we get started, let's talk ground rules. We are here to help Tyler work on goals for his diabetes. We are going to be respectful of each other and Tyler's privacy. Let's focus on opportunities to improve Tyler's T1D management.

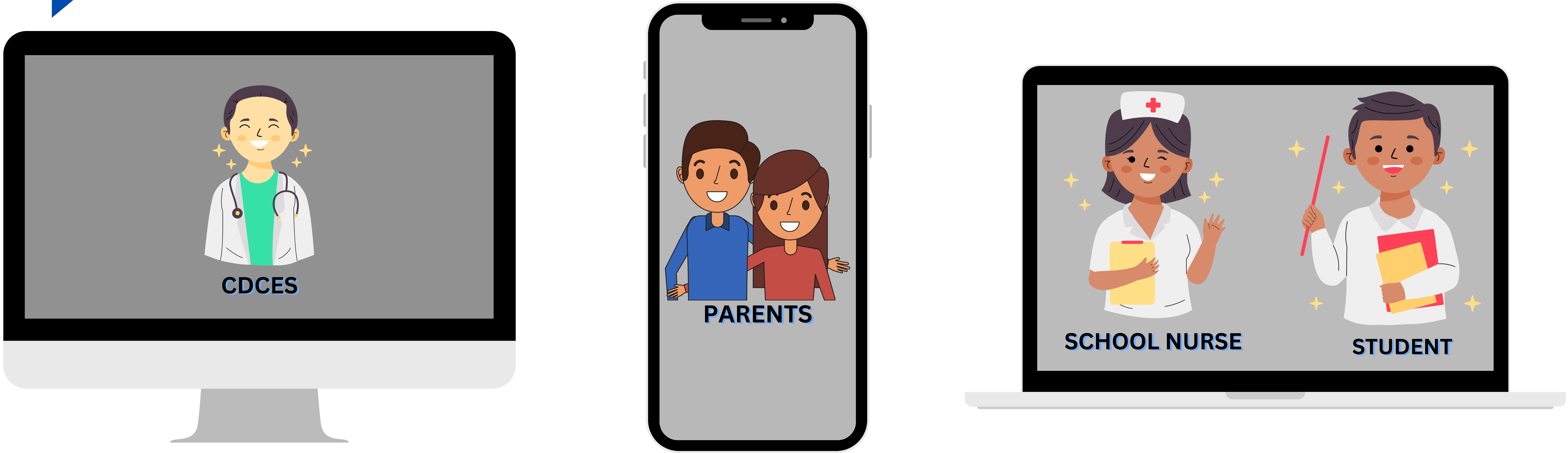

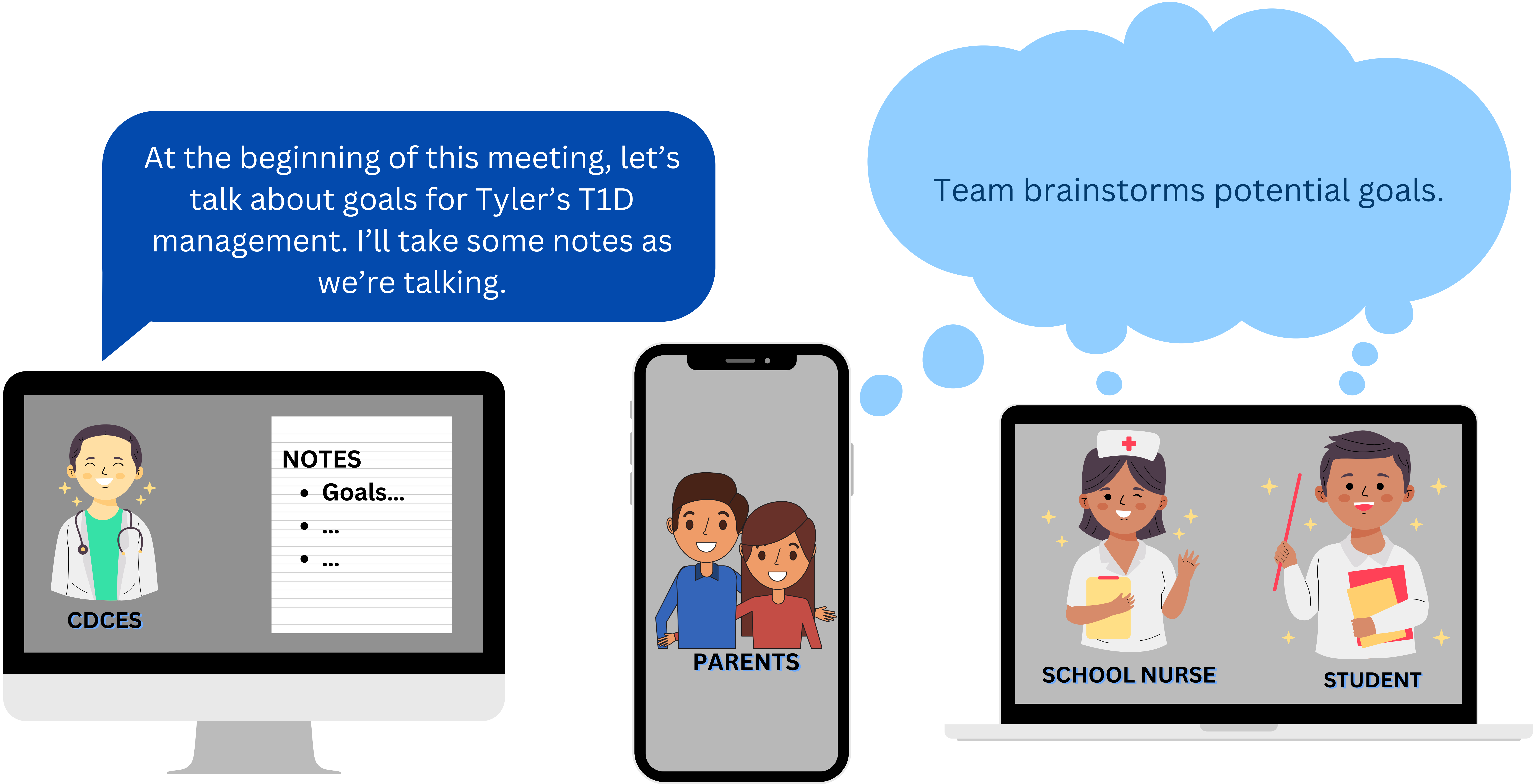
At the beginning of this meeting, let's talk about goals for Tyler's T1D management. I'll take some notes as we're talking.
Team brainstorms potential goals.
CDCES
NOTES
• Goals...
• ...
• ...
PARENTS
SCHOOL NURSE
STUDENT

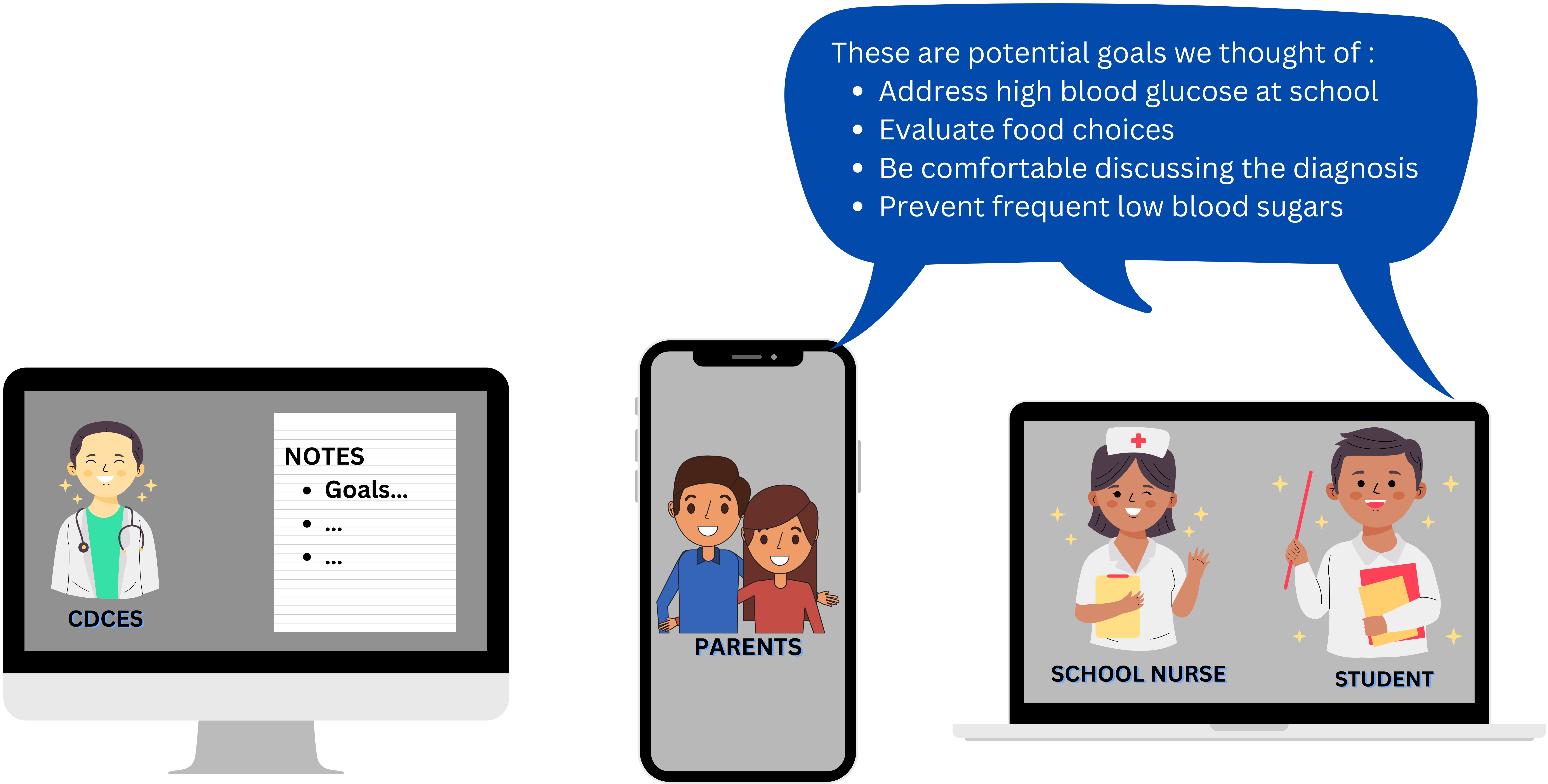
These are potential goals we thought of :
• Address high blood glucose at school
• Evaluate food choices
• Be comfortable discussing the diagnosis
• Prevent frequent low blood sugars
NOTES
• Goals...
• ...
• ...
CDCES
PARENTS
SCHOOL NURSE
STUDENT

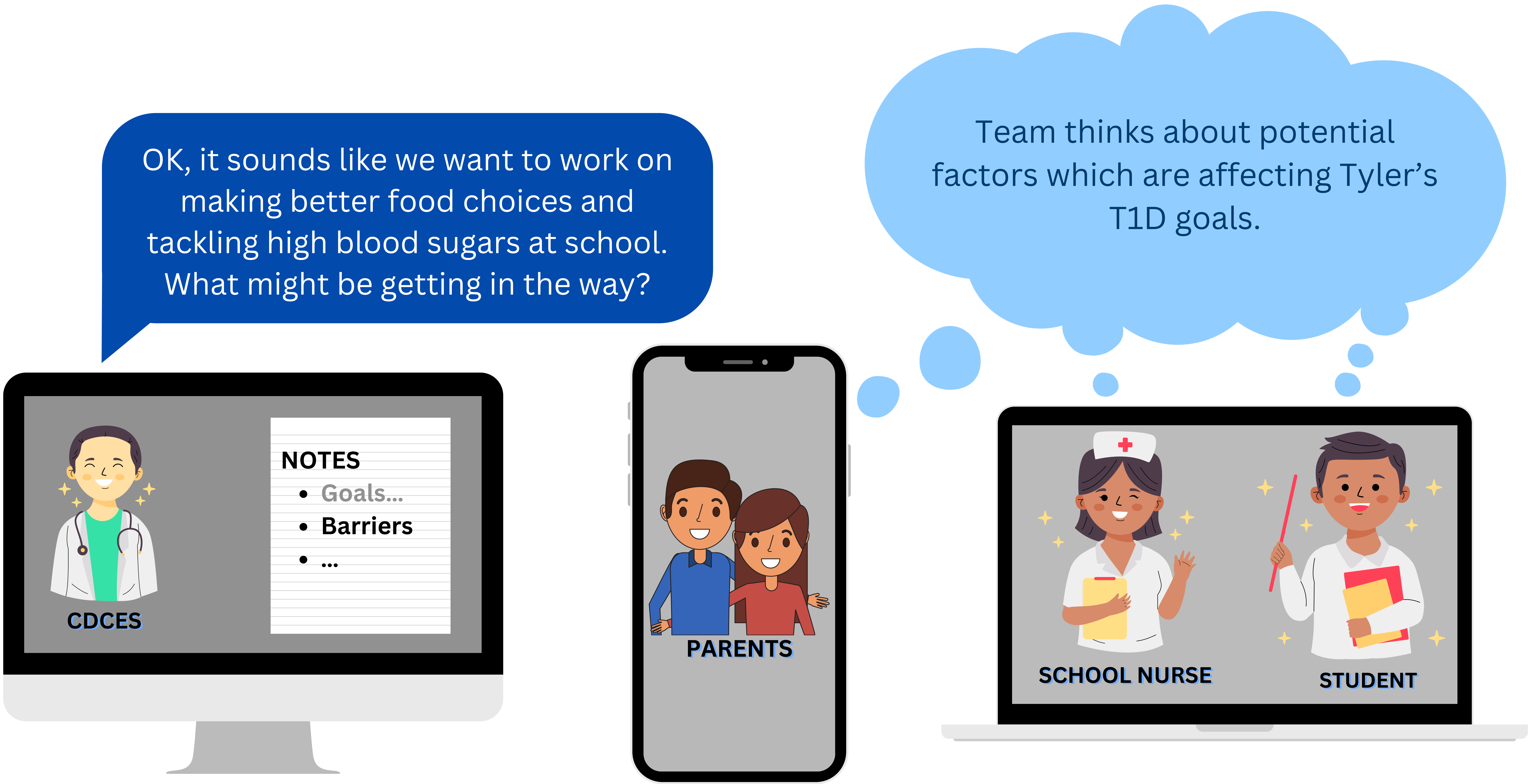
OK, it sounds like we want to work on making better food choices and tackling high blood sugars at school. What might be getting in the way?
Team thinks about potential factors which are affecting Tyler's T1D goals.
NOTES
Goals...
Barriers
...
CDCES
PARENTS
SCHOOL NURSE
STUDENT

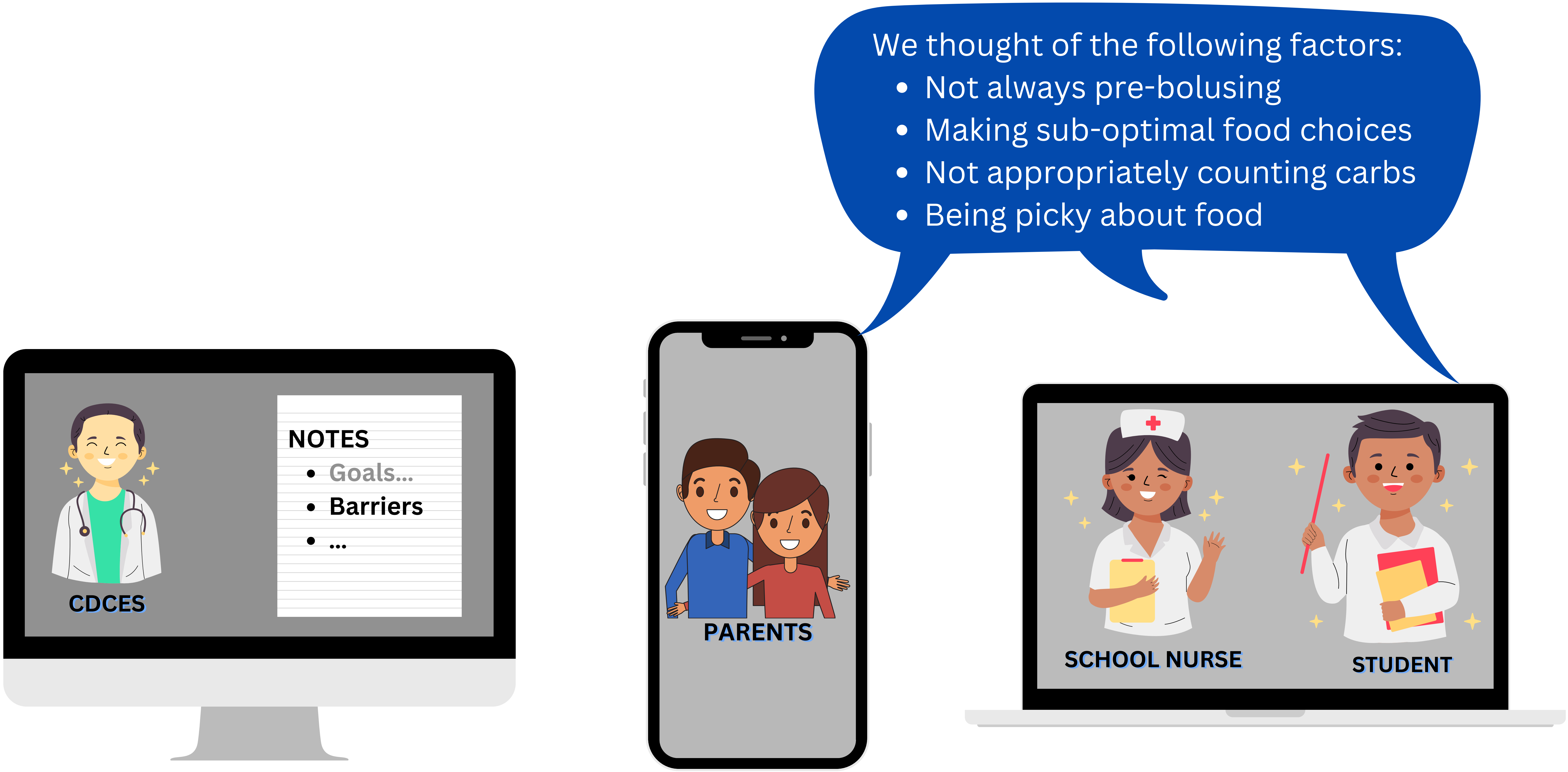
We thought of the following factors:
• Not always pre-bolusing
• Making sub-optimal food choices
• Not appropriately counting carbs
• Being picky about food
CDCES
NOTES
• Goals...
• Barriers
• ...
PARENTS
SCHOOL NURSE
STUDENT

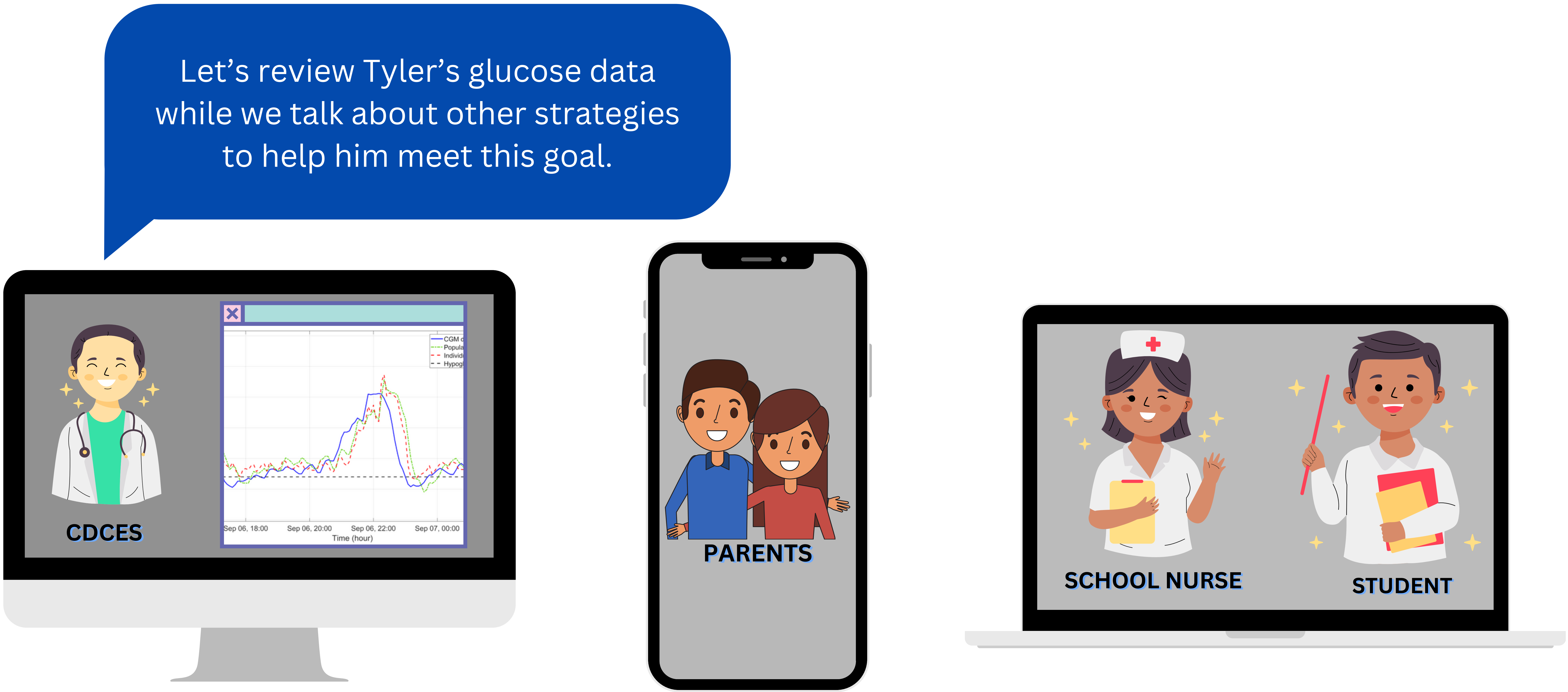
Let’s review Tyler’s glucose data while we talk about other strategies to help him meet this goal.
CDCES
Sep 06, 18:00
Sep 06, 20:00
Sep 06, 22:00
Sep 07, 00:00
Time (hour)
PARENTS
SCHOOL NURSE
STUDENT

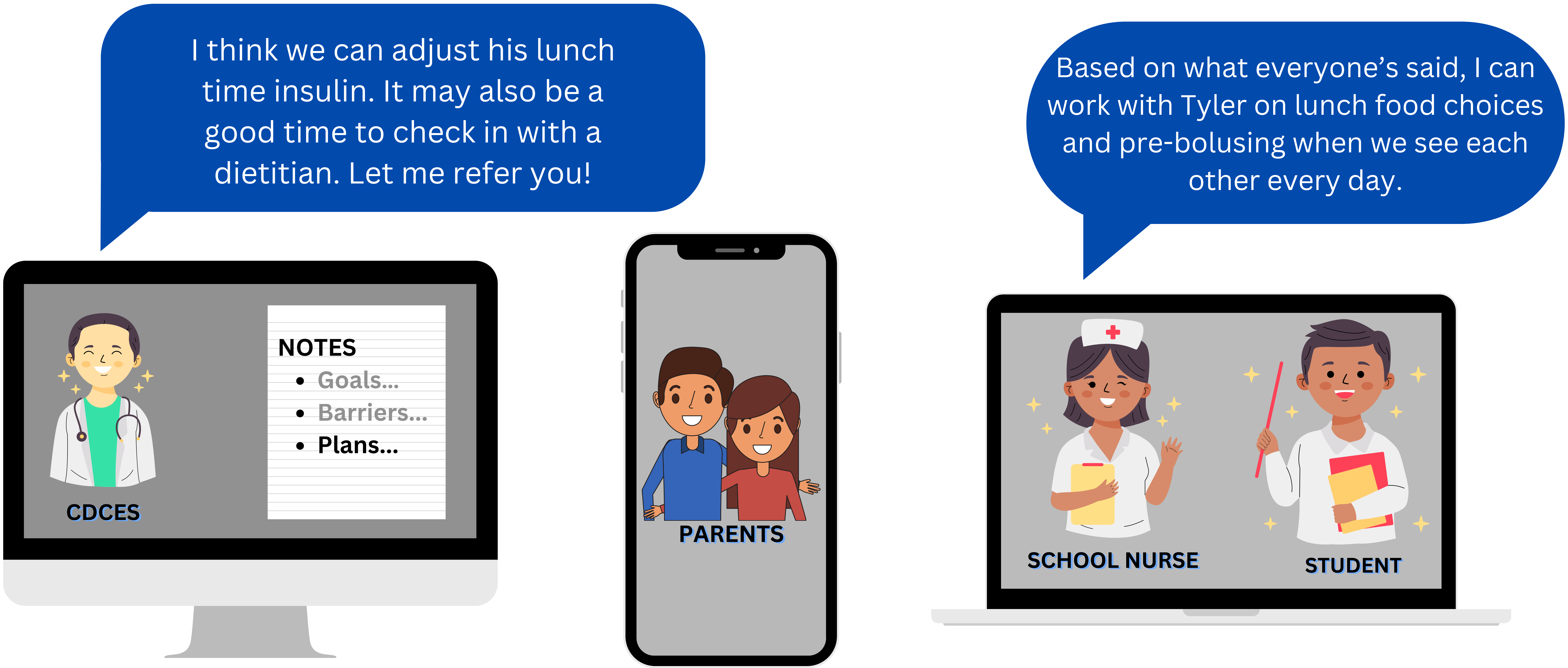
I think we can adjust his lunch time insulin. It may also be a good time to check in with a dietitian. Let me refer you!
NOTES
Goals...
Barriers...
Plans...
CDCES
PARENTS
Based on what everyone's said, I can work with Tyler on lunch food choices and pre-bolusing when we see each other every day.
SCHOOL NURSE
STUDENT

Here’s a list of other potential resources that may help Tyler or your family along the way! Let’s take a look at it. I will leave this list with you at the end of the meeting. Feel free to reach out and ask for any of those resources at your own time! Let’s zoom in and discuss what this additional support may look like for your family.

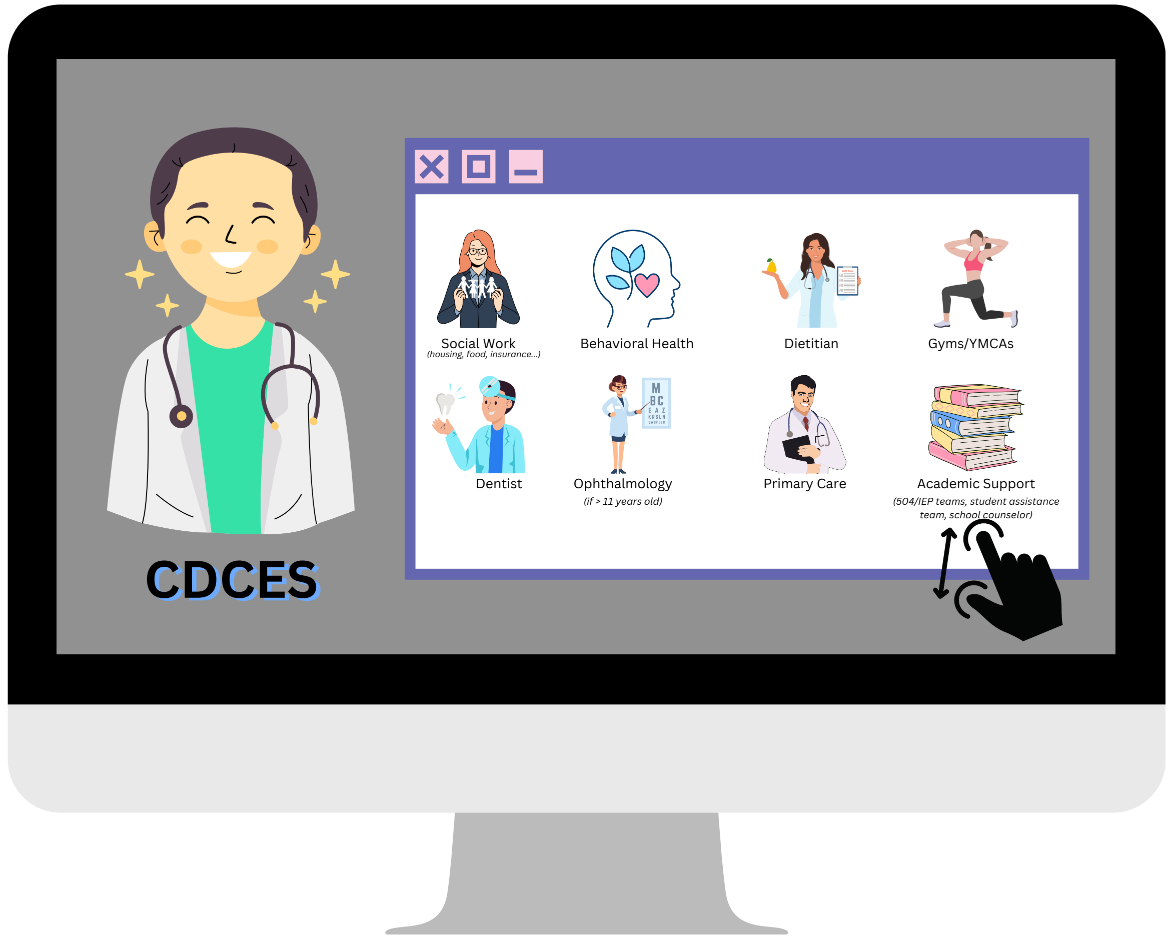

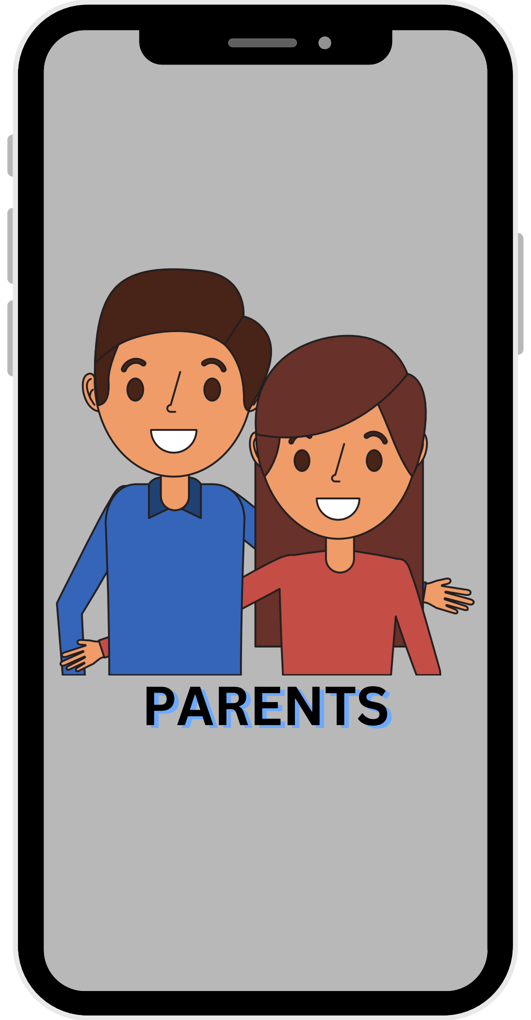

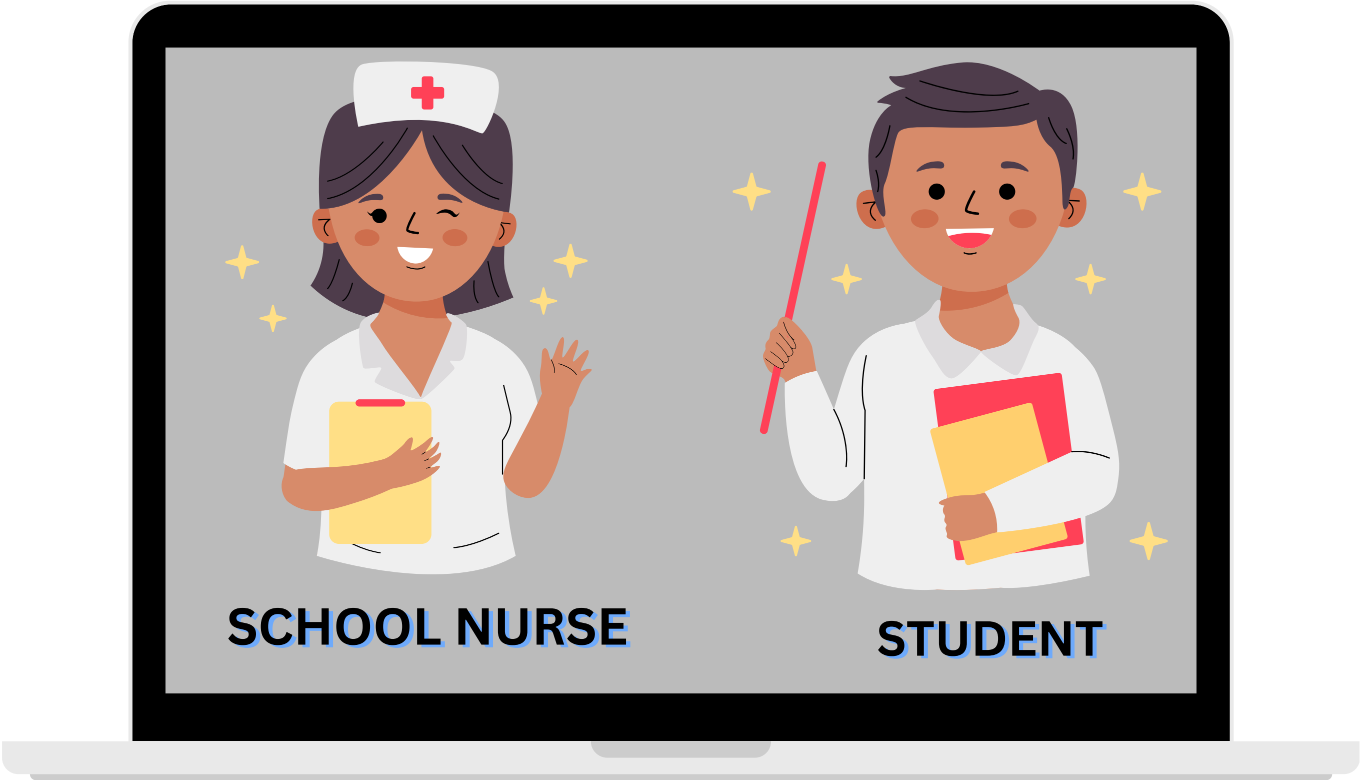

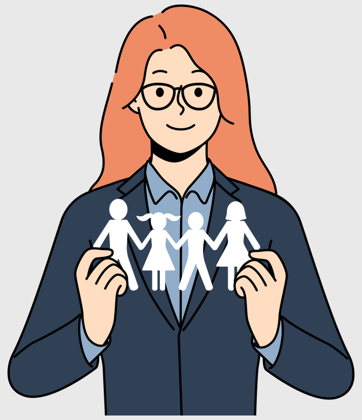

Social Work
(housing, food, insurance...)

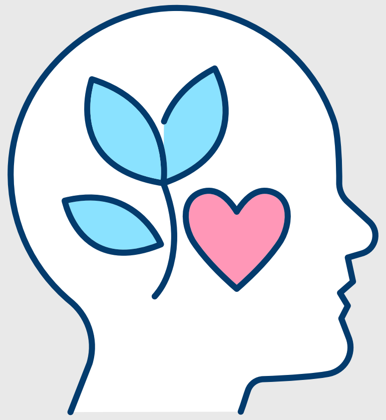

Behavioral Health

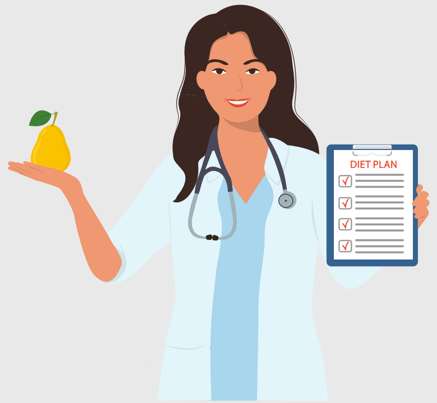

Dietitian

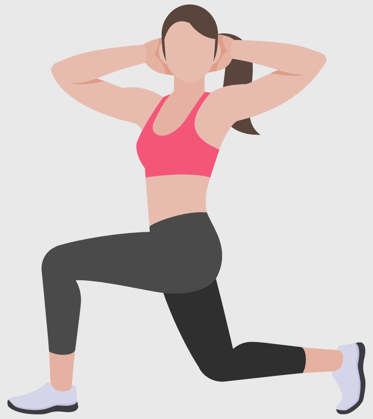

Gyms/YMCAs

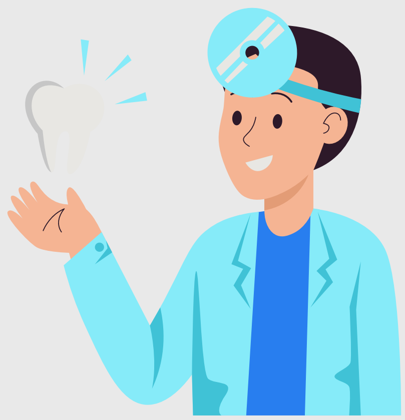

Dentist

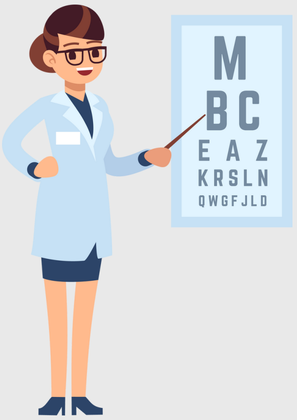

Ophthalmology
(if > 11 years old)

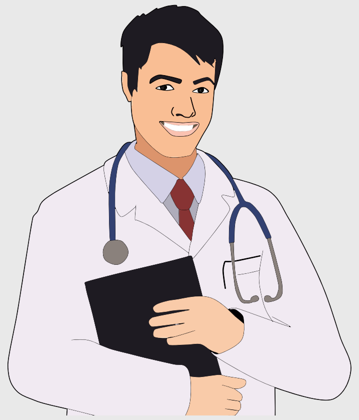

Primary Care

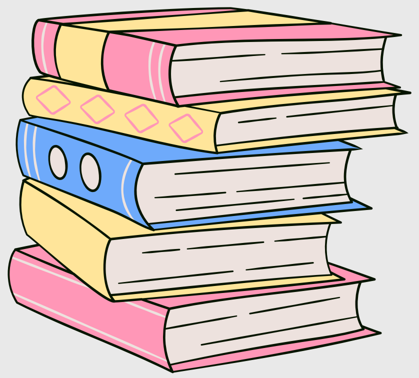

Academic Support
(504/IEP teams, student assistance team, school counselor)

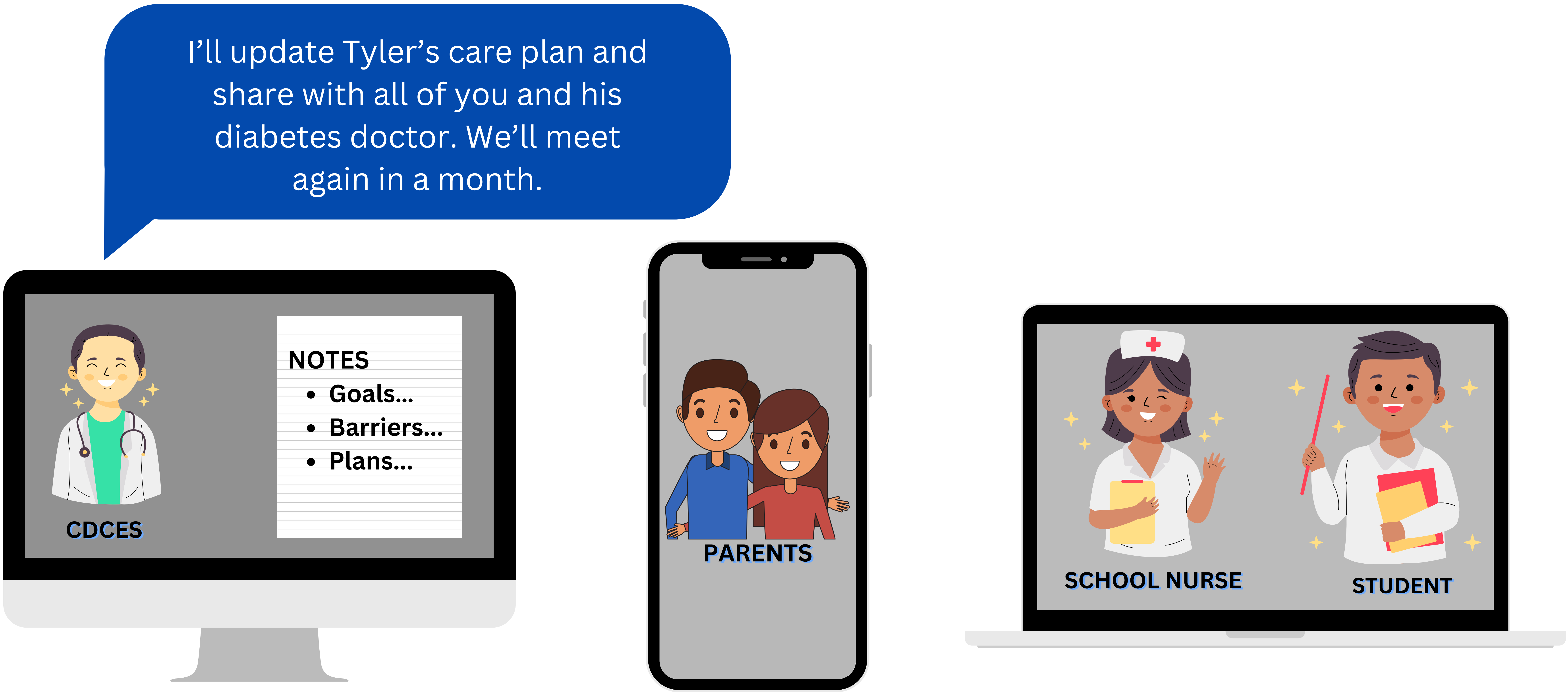
I'll update Tyler's care plan and share with all of you and his diabetes doctor. We'll meet again in a month.
CDCES
NOTES
Goals...
Barriers...
Plans...
PARENTS
SCHOOL NURSE
STUDENT

In planning for our future meetings, let’s think about the best way to tackle your family’s concerns. We would like for this process to be as flexible as possible. If you feel like you might want to bring up concerns privately, we can arrange for that. Similarly, I will regularly check in with Tyler to make sure he also gets the space to freely share some of his concerns!

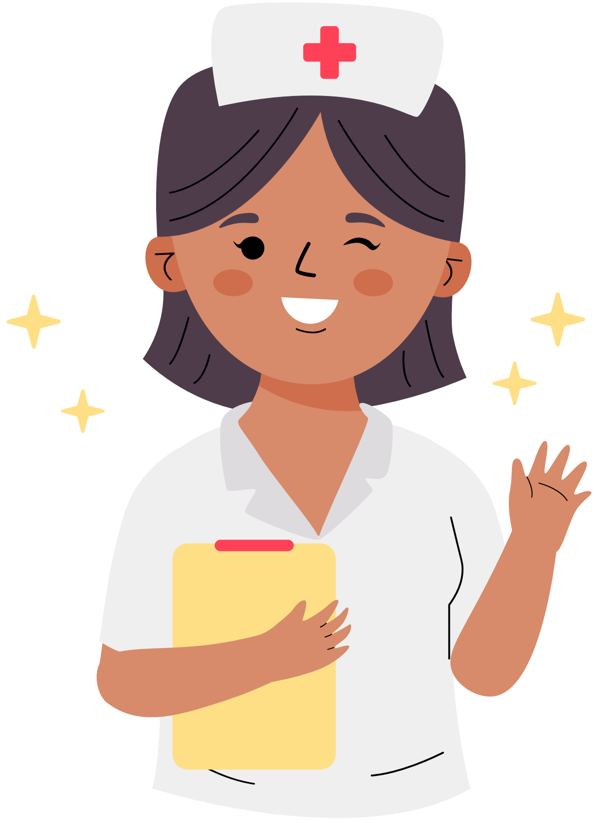

SCHOOL NURSE

We would really appreciate that! There are some matters we prefer not to discuss in front of Tyler, but we could really use your support!

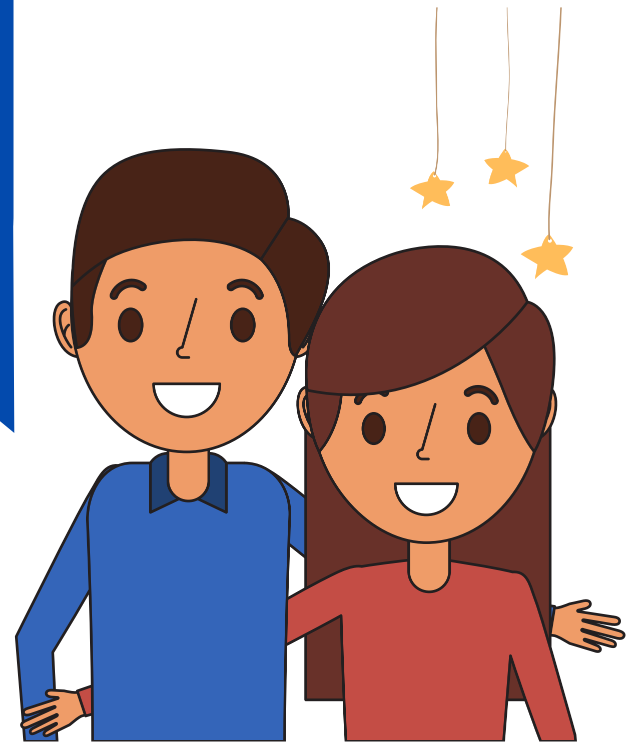

PARENTS

Between Meetings at home

Between meetings, we will:

- Help Tyler with his goals at home
- Follow-up with the dietitian as recommended
- Fill out some surveys about how we think Tyler is doing

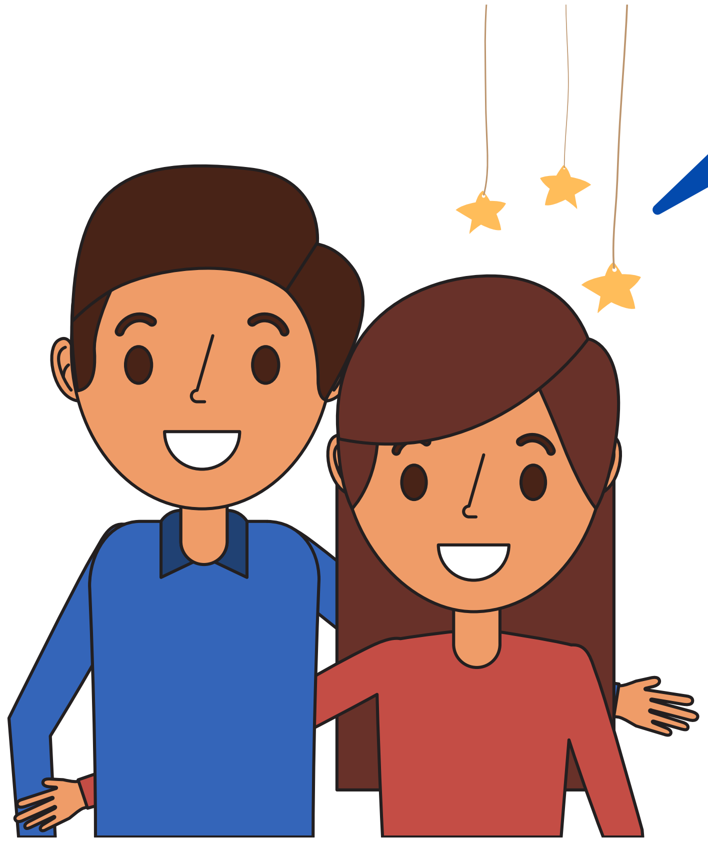

PARENTS

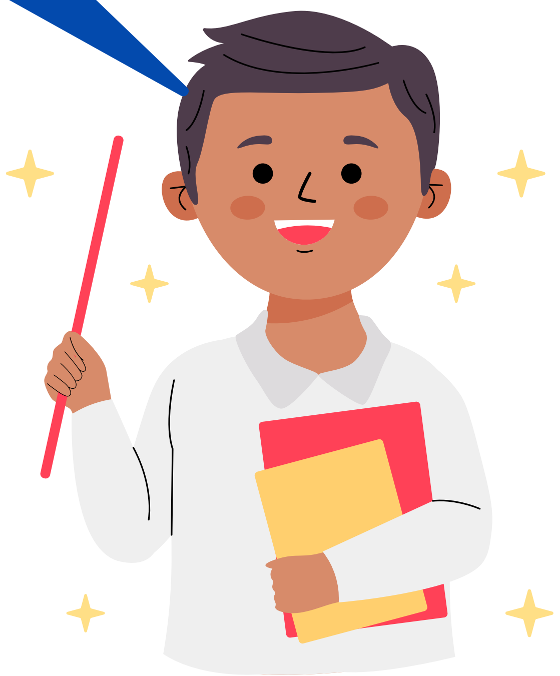

STUDENT

Between Meetings at school

Between meetings, I will:

- Help Tyler with his goals at school
- Keep track of Tyler's unscheduled visits to my health office for help with diabetes (e.g., ketones or hypoglycemia) & share back the information with the team
- Continue to assess Tyler's diabetes care at school and needs to identify new goals

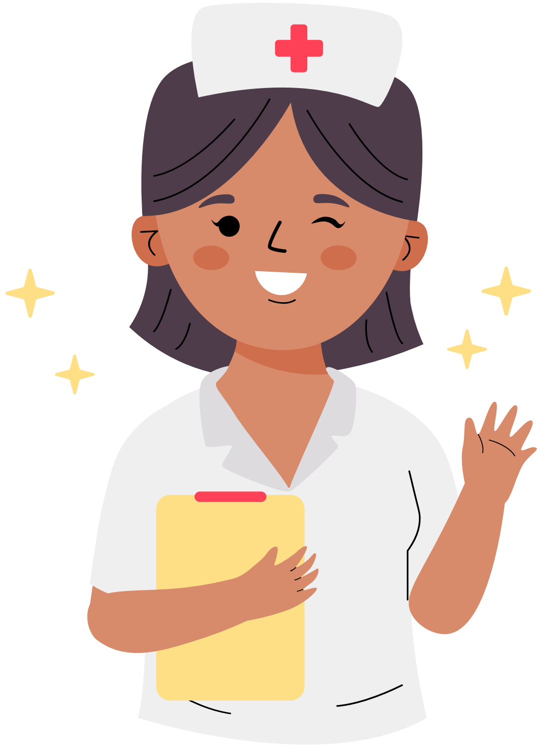

SCHOOL NURSE

To assess how Tyler is doing in SPACE, we will look at a set of clinical and school-related outcomes and track his progress! Here are a few examples:

Outcomes to track clinically

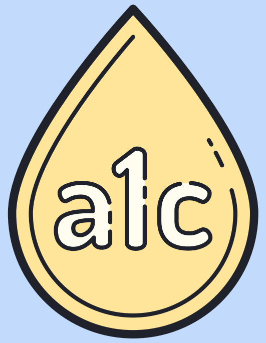

at clinic visits

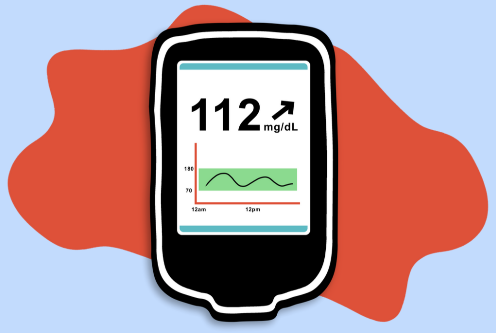

monthly CGM/glucometer data

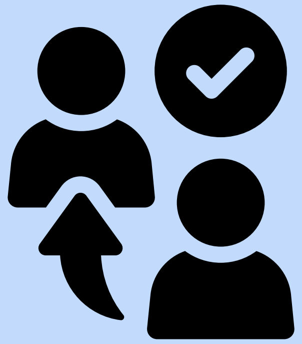

referrals made/scheduled/completed

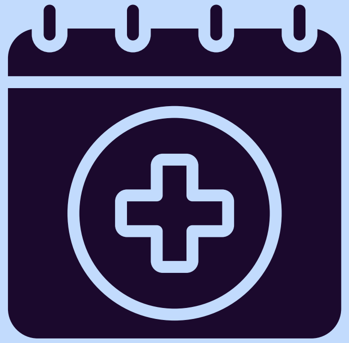

appointment attendance

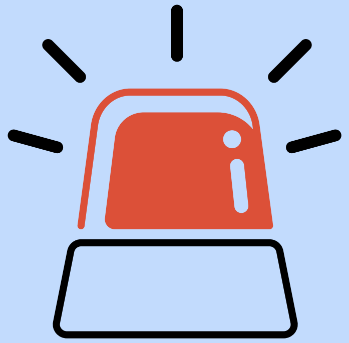

diabetes related ER/admissions

Outcomes to track at School

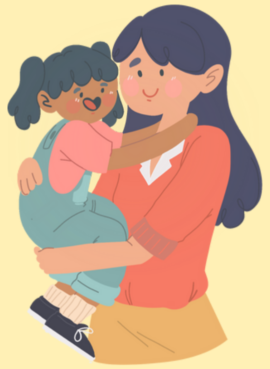

diabetes quality of life/distress

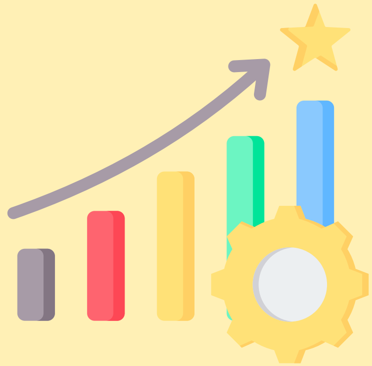

capability for self-management

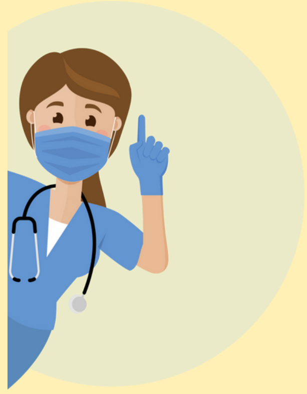

unscheduled school nurse visits and related communication

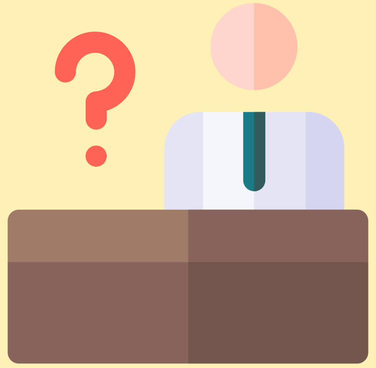

diabetes related absences/tardiness/ dismissals

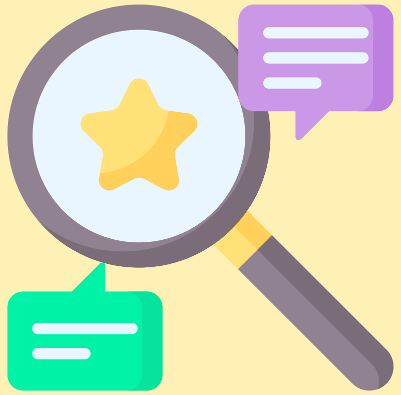

personal experience in SPACE

Today’s assignment - recap

In today’s assignment, we enrolled Tyler in SPACE and walked through what his first meeting would look like.
We helped him with his diabetes care goals, needed referrals and care coordination!

Just like Tyler, every student that enrolls in SPACE will have a personalized and targeted set of goals, referrals and outcomes to track and achieve!
